# Thermal proteome profiling (TPP) reveals NAMPT as the anti-glioma target of phenanthroindolizidine alkaloid PF403

**DOI:** 10.1016/j.apsb.2025.02.027

**Published:** 2025-02-26

**Authors:** Fangfei Li, Zhaoxin Zhang, Qinyan Shi, Rubing Wang, Ming Ji, Xiaoguang Chen, Yong Li, Yunbao Liu, Shishan Yu

**Affiliations:** State Key Laboratory of Bioactive Substance and Function of Natural Medicines, Institute of Materia Medica, Chinese Academy of Medical Sciences & Peking Union Medical College, Beijing 100050, China

**Keywords:** Glioma, Phenanthroindolizidine alkaloid, Thermal proteome profiling, NAMPT, Chemical biology, Target identification, PF403, CETSA

## Abstract

Glioma is difficult to treat due to the unique tumor microenvironment and blood–brain barrier. (13a*S*)-3-Hydroxyl-6,7-dimethoxyphenanthro[9,10-*b*] indolizidine (PF403), a phenanthroindolizidine alkaloid, has been identified as a promising therapeutic agent for the treatment of glioma. However, the anti-glioma mechanism of PF403 *in vivo* has not been conclusively verified and must be further elucidated. Hence, a strategy without chemical modification was applied to identify the target of PF403. In this study, we identified nicotinamide phosphoribosyl transferase (NAMPT) as the target of PF403 by using thermal proteome profiling (TPP). Moreover, microscale thermophoresis (MST), surface plasmon resonance (SPR), and isothermal titration calorimetry (ITC) experiments confirmed that NAMPT exhibits good affinity for PF403. Direct and indirect enzyme activity assays revealed that PF403 inhibited the catalytic activity of NAMPT, leading to a decrease in the concentration of nicotinamide adenine dinucleotide (NAD^+^) in U87 cells. X-ray diffraction and amino acid spot mutation experiments revealed that PF403 primarily relies on the formation of pi–pi interactions with residue Tyr188 to maintain binding with NAMPT (PDB code 8Y55). After NAMPT was knocked down with lentivirus, PF403 lost or partially lost its antitumor activity at the cellular and animal levels. These findings suggest that PF403 exerts antitumor activity by directly targeting NAMPT.

## Graphical abstract

The phenanthroindolizidine alkaloid PF403 was identified to directly target NAMPT *via* thermal proteome profiling (TPP), and subsequent validation of the interactions and biological functions was performed.

**Figure:**
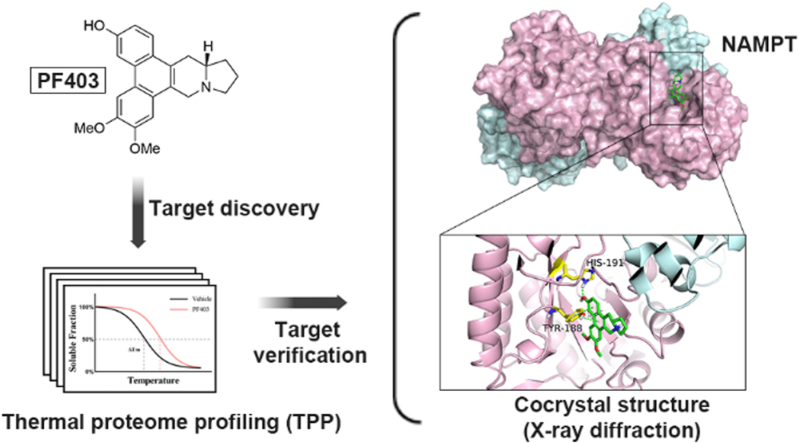

## 1 Introduction

Gliomas account for almost 30% of primary brain tumors and 80% of malignant tumors and are responsible for most deaths caused by primary brain tumors^1^. These glioma tumors are characterized by rapid growth, invasion into surrounding brain tissue, and a high recurrence rate^1^^,^^2^. Glioblastoma, the most malignant type of glioma, has a median overall survival of 15–17 months and a five-year survival rate of less than 5%^3^^,^^4^. Moreover, glioma cells exhibit a high degree of resistance to conventional therapeutic agents, including the first-line chemotherapy drug temozolomide (TMZ), which inevitably leads to tumor recurrence and poor prognosis^5^^,^^6^. Therefore, further research and innovative drugs or therapies are urgently needed to improve the outcomes and quality of life of individuals affected by this torturous disease^7^.

**Figure 1:**
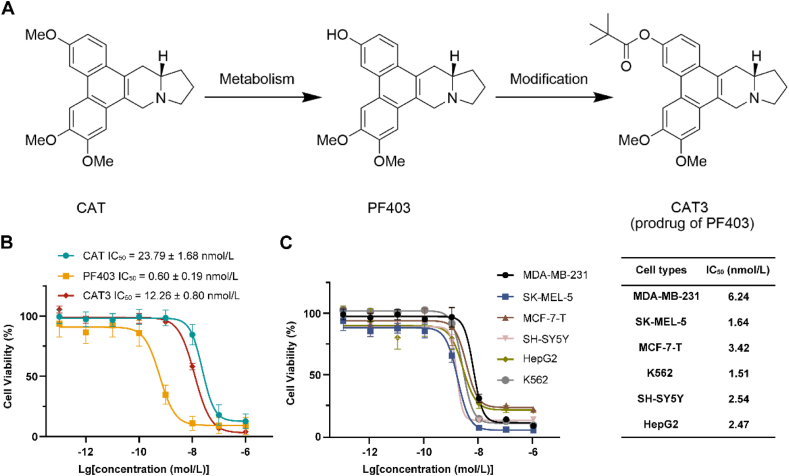
Structures of small molecules and their antitumor activities. (A) Structures of the phenanthroindolizidine alkaloid CAT, its metabolite PF403, and CAT3 (prodrug of PF403). (B) U87 proliferation was inhibited by treatment with CAT, PF403, and CAT3 according to the results of the cell counting kit-8 (CCK-8) assay (72 h). CAT: *n* = 9, PF403: *n* = 12, CAT3: *n* = 3. (C) Inhibitory activities of PF403 against a variety of malignant tumor cells. MDA-MB-231: *n* = 5, other cell lines: *n* = 6. All data are expressed as the mean ± SD.

Natural products have been widely used in the discovery of new drugs and are important sources for new drug targets due to their rich chemical diversity and pharmacological activity^8^. However, the mechanisms underlying the pharmacological activity of natural products are often difficult to determine, which limits the application and development of these compounds^9^. Therefore, identifying and verifying potential targets for natural products is highly important and can help reveal their mechanisms of action in organisms, leading to better knowledge of their pharmacological activities^10^^,^^11^. For example, through the TPP method, the natural product viopolide A was identified to target nucleolar protein 14, a crucial component for ribosome biogenesis, thereby exhibiting antitumor activity^12^. By clarifying the in-depth interactions between natural products and targets, enlightening information can be attained to guide the design and development of new drugs^13^.

(+)-Deoxytylophorinine (CAT; Fig. 1A) was obtained from the roots of *Tylophora atrofolliculata* and *Tylophora ovata* and exhibited potent *in vivo* antitumor effects^14^^,^^15^. These findings demonstrated the ability of CAT to effectively cross the blood–brain barrier, resulting in significant therapeutic effects on glioma^16^^,^^17^. During the investigations on its metabolic products *in vivo*, researchers discovered a compound named (13*aS*)-3-hydroxyl-6,7-dimethoxyphenanthro[9,10-*b*]indolizidine (PF403; Fig. 1A), which displayed exceptional antiproliferative activity with an IC_50_ value of less than 1 nmol/L; this finding was observed in various cancer cell lines, including TMZ-resistant cell lines^16^^,^^18^. Researchers designed and synthesized (13*aS*)-3-pivaloyloxyl-6,7-dimethoxyphenanthro[9,10-*b*] indolizidine (CAT3; Fig. 1A) as a prodrug of PF403 to improve its poor pharmacokinetic properties and rapid elimination^17^^,^^19^^,^^20^. However, there is controversy regarding the cellular targets of phenanthroindolizidine alkaloid, and several alternative mechanisms have been proposed, including interrupting the Hedgehog signaling pathway^17^, occupying the kinase binding domain of AKT to inactivate its function^21^, interfering with AKT/mTOR and AMPK signalling^22^, and modulating the p38 MAPK signaling pathway^23^. In addition, these alkaloids intercalate between DNA base pairs and inhibit the enzymes that are involved in the synthesis of DNA to exert antitumor effects^15^^,^^24^. Targeting RNAs can enable these alkaloids to exert antitumor and antiviral effects^14^^,^^25^. However, these studies have not been extensively validated with knockout target genes, particularly at the animal level.

Therefore, in this study, a series of experiments were performed to determine the mechanism that underlies PF403 activity. We determined U87 intracellular thermal profiles for up to 4000 proteins and compared the differences in the presence or absence of PF403. By the thermal proteome profiling-temperature range (TPP-TR) approach, PF403 was found to bind to NAMPT, which was subsequently confirmed by performing X-ray diffraction with protein–ligand complexes (PDB code 8Y55). Functionally, we performed direct and indirect enzyme activity and catalytic function assays. NAMPT in the U87 cell line was subsequently knocked down to further confirm that NAMPT was an important functional target of PF403 at the cellular level and in animal models (orthotopic and ectopic xenografts).

## 2 Materials and methods

### 2.1 Chemicals and reagents

CAT (C_23_H_25_NO_3_, M = 363.46), PF403 (C_22_H_23_NO_3_, M = 349.43), and CAT3 (C_27_H_31_NO_4_, M = 433.55) were synthesized in our laboratory and confirmed by ^1^H NMR, ^13^C NMR and MS analysis^26^^,^^27^. RIPA buffer, protease and phosphatase inhibitors, penicillin and streptomycin, PBS, 0.25% trypsin–EDTA, and a BCA protein assay kit were purchased from Thermo Scientific. DMEM, RPMI-1640, 5 × SDS-PAGE sample buffer, and TBST were obtained from Solarbio. Fetal bovine serum was purchased from Procell. Ultracentrifugal filter tubes and PVDF membranes were obtained from Merck Millipore. High-affinity Ni-NTA resin was purchased from GenScript. ECL chemical luminescence reagents were obtained from Applygen. A CCK-8 assay kit was purchased from TargetMol. Primary and secondary antibodies were obtained from Proteintech. Skim milk was obtained from Becton Dickinson and Company. Temozolomide was obtained from Aladdin. FK866 was purchased from Beyotime.

### 2.2 Cell culture

MDA-MB-231, SK-MEL-5, MCF-7, SH-SY5Y, HepG2, and K562 cells were purchased from the Peking Union Medical College Cell Bank (Beijing, China). The human brain astrocytoma U87 cell line was purchased from Procell Co., Ltd. High-glucose DMEM, RPMI-1640 medium, phosphate-buffered saline, 0.25% trypsin–EDTA, penicillin, streptomycin, and fetal bovine serum were used for cell culture and proliferation. The cell lines were cultured in high-glucose DMEM or RPMI-1640 supplemented with 10% FBS, 100 U/mL penicillin, and 100 g/mL streptomycin. The culture environment for the cells was 37 °C and 5% CO_2_.

### 2.3 Cell viability assay

The effects on cell proliferation were measured using a CCK-8 assay kit. A total of 3 × 10^3^ cells were seeded per well in 96-well plates and incubated for 24, 48, or 72 h after drug administration. After 10 μL of CCK-8 solution was added to each well and incubated at 37 °C for 1–2 h, the absorbance at OD_450_ was read with an enzyme marker. The data were recorded and analyzed by GraphPad Prism software (version 8.0.2). For the cell growth assay, 2 × 10^3^ cells were seeded per well in 96-well plates (*n* = 5). Ten microlitres of CCK-8 reagent was added at a fixed time every day, and the OD_450_ was read after 2 h. The statistical data were analyzed, and cell growth curves were plotted after a continuous period of 7 days.

### 2.4 Thermal protein profiling (TPP)

*In vivo* TPP experiments were performed in accordance with the methods of previous reports, with only minor modifications^12^^,^^28^^,^^29^. U87 cells (1 × 10^7^) were treated with 10 μmol/L PF403 or the same volume of DMSO as the vehicle control. After the cells were incubated at 37 °C for 3 h, they were collected and washed with PBS. Glioma cells were resuspended at a volume concentration of 1 × 10^7^/200 μL in PBS containing protease and phosphatase inhibitors. The cell suspension (200 μL) was then divided into ten equal portions (10 × 20 μL) corresponding to ten temperatures (37, 41, 44, 47, 50, 53, 56, 59, 63, and 67 °C). These samples were first heated at the corresponding temperature for 3 min and cooled at room temperature for 3 min. Then, 0.1% NP40 in PBS (80 μL) was added, the samples were frozen and thawed in liquid nitrogen three times, and centrifugation was performed at 15,000 rpm for 30 min (5425R, Eppendorf, Hamburg, Germany) to obtain the supernatant. The protein concentrations of the 37 °C samples were determined by a BCA kit, and 25 μg of protein was added to a 10 kDa ultrafiltration tube (the same volume of 41–67 °C samples was collected). DTT (100 mmol/L DTT configured with 8 mol/L urea) and IAA (20 mmol/L IAA configured with 50 mmol/L NH_4_HCO_3_) were used to reduce the protein concentrations in the samples. After displacement to a 100 mmol/L TEAB system, the protein samples were digested with trypsin at 37 °C for 16 h. Peptide labeling was conducted by TMT10 reagents (Thermo Fisher Scientific, 90110, USA) following the instructions, and hydroxylamine (5%, 8 μL) was applied to quench the reaction. Samples with labeled peptides at 10 temperature points were combined into DMSO and PF403 groups and dried under vacuum centrifugation for subsequent peptide fractionation. Peptide fractionation was performed exactly as described in the literature. Each group was divided into 18 samples, spin-dried, and resuspended in 10 μmol/L ddH_2_O, which were then injected into the protein mass spectrometry for analysis.

### 2.5 LC–MS/MS analysis

LC–MS/MS analysis was performed on an Orbitrap Fusion™ Lumos™ Tribrid™ mass spectrometer coupled to an Ultimate 3000 system (Thermo Fisher Scientific, USA). Peptides were loaded onto a trap column (0.3 mm × 5 mm, C18, 5 μm, 100 Å) using ddH_2_O containing 0.1% formic acid at a flow rate of 10 μL/min. The peptides were then separated by an Acclaim PepMap RSLC analytical column (75 μmol/L × 15 cm, nanoViper C18, 2 μmol/L, 100 Å) at a column temperature of 40 °C. Mobile phase A consisted of 0.1% formic acid in water, while mobile phase B consisted of 0.1% formic acid in a mixture of 80% acetonitrile and 19.9% water. The gradient was as follows: 5 min of 4% B, 4%–30% B for 65 min, 30%–80% B for 5 min; 80% B for 5 min; 80%–4% B for 5 min, and 4% B for 5 min, 300 nL/min. The peptides were ionized by using a spray voltage of 2.4 kV and a capillary temperature of 320 °C. The instrument was set to data-dependent mode, automatically switching between MS and MS2 scans. Full scan MS spectra (*m*/*z* 350–1500) were acquired with a maximum injection time of 50 ms at a resolution of 60,000 and an automatic gain control (AGC) target of 4 × 10^5^ charges. High-resolution MS2 spectra were acquired in the Orbitrap with an exclusion time of 25 s, a maximum injection time of 30 ms at 15,000 resolution (isolation window *m*/*z* 1.6), an AGC target value of 5 × 10^4^, and a normalized collision energy of 30%. Only precursors with charge states between 2 and 7 were selected for fragmentation.

### 2.6 Database analysis and post-processing

The elucidation of data was analyzed by the Proteome Discoverer (2.3) workstation, which supports the modifications by TMT Reagents and the relative quantitation of reporter ions released from labeled peptides. Peptide sequences were determined by matching the protein database (Uniport, *Homo sapiens* (Human)) with the obtained fragmentation patterns by the SEQUEST HT algorithm, and protein abundances obtained from each analysis were normalized using TMT126 (samples treated at 37 °C) as a control to derive the following data: 126/126, 127N/126, 127C/126, 128N/126, 128C/126, 129N/126, 129C/126, 130N/126, 130C/126, 131/126. The above exported data was subsequently analyzed through the TPP R package from Bioconductor.

The analysis data obtained from the Proteome Discovery 2.3 software were imported to ProSAP (https://github.com/hcji/ProSAP). The two replicates of the control group and PF403 group were defined separately as ‘Rep 1 Ctrl’, ‘Rep 2 Ctrl’, ‘Rep 1 Case’, and ‘Rep 2 Case’, while all parameters were set as default. Finally, all screened proteins were sorted based on the overall score.

### 2.7 Cellular thermal shift assay

For the living cell CETSA experiment, cells were treated with or without PF403 (10 μmol/L) for 3 h. The cells were collected and equally divided into 12 parts with 0.2% NP40-PBS solution, heated at specific temperatures (37–72 °C), and then cooled for 3 min. The samples were lysed by freeze–thawing three times with liquid nitrogen. After ultracentrifugation, the supernatant was subjected to a WB assay. In addition, for the cell lysate and recombinant NAMPT CETSA experiments, the samples were divided into two parts and treated with or without PF403 (10 μmol/L) for 1.5 h at 25 °C. Subsequent manipulation was consistent with the above procedure except that the cells were not lysed. For concentration-dependent CETSAs, the samples (live cells, lysate, recombinant NAMPT) were divided into 10 parts. Subsequently, the 10 parts were treated with vehicle control or (2, 5, 10, 20, 50, 100, 200, 300, or 500 μmol/L) PF403, followed by heating for 3 min and cooling for 3 min. Then, high-speed centrifugation was performed to collect the supernatant for the WB assay.

### 2.8 Expression and purification of recombinant NAMPT and the mutants

The DNA coding sequences of human NAMPT (His-tagged) and the corresponding His-tag were subcloned and inserted into the pET-28a(+) (BGI-write) expression vector. The vectors were subsequently transformed into *E. coli* BL21 (DE3) cells for protein production. The *E. coli* were cultured until they reached an OD_600_ of 0.6–0.8. Afterwards, the cells were induced with 1 mmol/L IPTG at 18 °C and 180 rpm for 16–20 h (HZQ-F100, HAO CHENG, Taicang, China). A high-affinity Ni NTA purification medium was used to purify these recombinant proteins with Tris–HCl buffer. After the protein was fully enriched on Ni-NTA, the proteins were eluted using gradient buffer containing imidazole (0, 25, 50, 100, 200, and 400 mmol/L). The gradient-eluted proteins were stained with Coomassie Brilliant Blue to confirm purity. The samples were then concentrated in a 10 kDa centrifugal filtration tube.

### 2.9 MST analysis

Recombinant NAMPT was labeled with a Monolith Protein Labeling Kit (red-NHS 2nd Generation Kit). Subsequent pretest experiments were performed to test the labeling effect. Recombinant NAMPT was diluted to a working concentration of 40 nmol/L using an assay buffer. Small molecules are soluble in DMSO, and the DMSO content of the protein–ligand mixed solution must be less than 5%. Subsequently, a Monolith NT.115 (NanoTemper, Munich, Germany) was used for thermophoresis measurements.

### 2.10 SPR assay

The interaction between the recombinant proteins NAMPT and PF403 was quantified using a Biacore 1K (Cytiva, Sweden) with a CM7 sensor chip. The 300 mmol/L concentration of buffer salts and 0.1% BSA were used to reduce the non-specific binding on the chip. The amine coupling method was applied to immobilize recombinant NAMPT on the Sencor chip. NAMPT coupling was performed at a concentration of approximately 27.5 μg/mL in a pH 5.0 sodium acetate solution, with a chip activation time of 420 s and a closure time of 420 s. Small molecules were solubilized with 5% DMSO, and PBST was used as the running buffer. Biacore Insight Evaluation (5.0.18.22102) was used to calculate the kinetic parameters of *K*_d_.

### 2.11 ITC titration

The ITC experiment was performed on the nanoITC (TA-USA). PF403 was solubilized in PBS buffer with 2% DMSO, and the protein solution was replaced with the same buffer. The stirring speed was set to 300 rpm (nanoITC, TA Instruments, New Castle, DE, USA), and the titration interval was 150 s/15 drops at 25 °C. Raw data were analyzed with NanoAnalyze DataAnalysis (version 3.12.5).

### 2.12 NanoDSF assay

Label-free nanoDSF technology operated by Prometheus NT.48 (NanoTemper, Munich, Germany) can accurately detect changes in endogenous fluorescence during thermal and chemical denaturation of proteins. Control proteins and protein–ligand complexes were pipetted into the sample rack with 8–10 μL of sample. The temperature increase interval was 20–95 °C, and the temperature increase rate was 1 °C/min.

### 2.13 Indirect NAMPT activity *in vitro*

The inhibition of NAMPT activity was evaluated using a NAMPT activity assay kit (Abcam, ab221819). The assay is based on a multistep reaction that converts WST-1 to WST-8 formazan. Formazan was easily detected at an OD of 450 nm. The inhibitory effect of PF403 on recombinant NAMPT was determined by a two-step method. Reaction mix I included NAMPT assay buffer, ATP, NMNAT1, nicotinamide, PRPP, and ddH_2_O. Reaction Mix II included WST-1, ADH, diaphorase, and ethanol solution. Solvent controls and inhibitor controls were used for comparison with the inhibitor test samples. Reaction Mix I was incubated at 30 °C for 60 min, and then Reaction Mix II was added and mixed thoroughly. The optical density was measured at 450 nm on a microplate reader in kinetic mode every 5 min for at least 30 min at 30 °C protected from light.

### 2.14 Direct NAMPT activity assay *in vitro*

Direct inhibition of NAMPT activity was evaluated using a NAMPT inhibitor screening assay kit (BPS Bioscience, 71276-1, US). All the samples and controls were tested in duplicate. PF403 was diluted with 5% DMSO to concentrations between 10^−10^ and 10^−6^ mol/L and preincubated with recombinant NAMPT for 30 min. This kit was used to simulate the reaction between NAM and PRPP *in vivo* to generate PPi and NMN. Subsequently, NMN was quantified by fluorescence labeling through chemical reactions in a buffer solution.

### 2.15 Measurement of NAD ^+^ levels

NAD^+^ is a coenzyme found in all cells and consists of both NAD^+^ (oxidized) and NADH (reduced) forms. NAD^+^ can be used to transfer electrons during redox reactions and as a substrate used by many enzymes to participate in intracellular reactions. We used an NAD^+^/NADH assay kit (Beyotime, S0175, Shanghai, China) based on the WST-8 method to determine the NAD^+^/NADH content. Cells in 6-well plates were collected and lysed with NAD^+^/NADH extract on ice, and the supernatant was used as the sample to be tested. A control solution with a gradient concentration of NADH was prepared with a 10 mmol/L NADH standard. The supernatant from the sample was first aspirated and used to determine the total amount of NAD^+^ and NADH. Subsequently, the sample was heated at 60 °C for 30 min to remove the NAD^+^, after which the supernatant was collected to determine the NADH concentration. Ethanol dehydrogenase was added to each well, and the plate was incubated at 37 °C in the dark for 10 min. Then, 10 μL of chromogenic solution was added, and the plate was incubated for 30 min. Afterward, the absorbance at 450 nm was measured, and the content was calculated by comparison with the standard curve. The content of NAD^+^ was calculated by subtracting the content of NADH from the total amount.

### 2.16 Protein–ligand cocrystal culture and data collection

The crystals were grown by hanging drop vapor diffusion. The crystals were then rapidly immersed in a reservoir solution supplemented with 20% glycerol as a cryoprotectant, mounted on loops, and flash-cooled at 100 K in a nitrogen gas cryostream. Diffraction data were collected from a single crystal at the Shanghai Synchrotron Radiation Facility BL18U beamline in China, with a wavelength of 0.9793 Å at 100 K. The structures were solved using the molecular replacement method with the published NAMPT structure (PDB ID: 5UPE) as a search model^30^. Structure refinement was performed using PHENIX, and iterative manual modeling was performed using COOT^31^^,^^32^.

### 2.17 Molecular docking studies

Docking studies were performed with the Glide module within the Schrödinger package based on the united atom scoring function. The protein preparation wizard was utilized to prepare the crystal structures of NAMPT. For the cocrystal structure of the NAMPT–PF403 complex, we performed protein–ligand interaction analyses after protein preparation in Maestro. Images elucidating the protein–ligand interactions were produced by PyMOL (version 1.7.2.1).

### 2.18 Molecular dynamics simulation

The protein–ligand complexes were subjected to MD simulation with Desmond. First, the chosen complexes were embedded in a simple point charge water model after processing with the Protein Preparation Wizard. An orthorhombic box with a 10 Å radius was utilized to describe the core, and all the complexes were neutralized by adding numerous Na^+^ and Cl^–^ ions with the physiological salt concentration at 0.15 mol/L. Subsequently, the systems were subjected to a minimization job to relax with the maximum number of interactions set to 2000 and the convergence threshold set to 1.0 kcal/mol/Å. Notably, the OPLS_3 force field was applied to the protein–ligand systems, and the other parameters were set to their defaults. The minimized systems were subjected to MD for a simulation time of 200 ns with a time step of 2 fs in an NPT ensemble using a Nose-Hover thermostat at a temperature of 300 K and a pressure of 1.01325 bar set by Martyna-Tobia-Klein barostats to relax the complex. During the process of MD simulation, the energies were calculated every 1.2 ps, and every trajectory was recorded with a time interval of 4.8 ps. Moreover, the RMSD, root mean square fluctuation (RMSF), and intermolecular interactions were monitored.

### 2.19 Western blotting

U87 cells were collected by scraping and homogenized with RIPA buffer containing protease and phosphatase inhibitors. The supernatant of the soluble proteins was obtained by centrifugation at 15,000 rpm for 20 min at 4 °C (5425R, Eppendorf, Hamburg, Germany). The protein concentration was determined by a BCA Protein Assay Kit (Pierce, Waltham, MA, USA), and the concentration of each group was adjusted to the same level. Then, 5 × SDS-PAGE sample buffer was added to the samples, which were boiled at 100 °C for 10 min. The samples were separated *via* 10% SDS-PAGE and subsequently transferred to PVDF membranes. Subsequently, the membranes were blocked with 5% skim milk in TBST at room temperature for 1.5 h, incubated at 4 °C overnight with the desired primary antibodies, and washed three times with TBST. The membranes were incubated with the corresponding secondary antibodies at room temperature for 1.5 h. After the ECL chemical luminescence substrate was added, the proteins were visualized *via* a Tanon-5200 (Tanon, Shanghai, China) chemical luminescence imaging system and analyzed *via* ImageJ 1.53 software.

### 2.20 shRNA transfection by lentivirus

Pre-experiments were first carried out to determine the optimum concentration of puromycin for U87 cells at 2 mg/mL (the concentration that killed more than 90% of normal cells at 48 h). We employed the 1/2 small-volume transfection method and determined that the multiplicity of infection (MOI) was 20, while the addition of polybrene (5 μg/mL) was used to increase the transfection efficiency. Seventy-two hours after transduction, puromycin was added to the culture medium to eliminate stable shRNA clones. The three shRNA sequences used to construct NAMPT are shown below.

#1: AGCGATAGCTATGACATTTATCTCGAGATAAATGTCATAGCTATCGCT.

#2: GTGAAGATCTAAGACATTTAACTCGAGTTAAATGTCTTAGATCTTCAC.

#3: TCAGCGATAGCTATGACATTTCTCGAGAAATGTCATAGCTATCGCTGA.

### 2.21 Animal studies

Six-week-old female athymic nude mice (BALB/c-nude) were obtained from GemPharmatech (Beijing, China). U87 cells (3 × 10^6^ cells per animal) were injected into the rear right armpit of nude mice to generate xenograft tumors. Seven to ten days after injection, the tumors were visible to the naked eye (approximately 100 mm^3^), and the mice were randomized into five groups (12 mice per group). The PF403 treatment groups were administered different doses of PF403 once daily *via* intragastric gavage (low dose, 3 mg/kg; median dose, 6 mg/kg; high dose, 12 mg/kg). The positive control drug used was temozolomide (50 mg/kg, i.g., Days 1–5), the first line of glioma treatment, while the control group was treated with 0.5% CMC-Na solution as a control. To validate the NAMPT knockdown group, we established the following groups: a control group (vehicle control, i.g., once daily, *n* = 12), a shNAMPT-2 knockdown group (vehicle control, i.g., once daily, *n* = 12), and a shNAMPT-2 knockdown group treated with CAT3 (6 mg/kg, i.g., once daily, *n* = 12). The body weights of the nude mice were recorded every two days, and the tumor weights were recorded on the 14th day after the mice were sacrificed.

For the orthotopic glioma model, six-week-old female athymic nude mice (BALB/c-nude) were obtained from GemPharmatech (Beijing, China). Anesthetize nude mice with tribromoethanol at a concentration of 20 mg/mL and a dosage of 0.2 mL/10 g body weight. U87 cells (shNAMPT-2, 5 × 10^5^ cells) suspended in 5 μL PBS buffer were injected intracranially into nude mice using stereotaxic restraint. The location was bregma +1.0 mm, right lateral 2.0 mm, profundity 3.5 mm, and return to 3.0 mm. After 3 days, 6 mg/kg of CAT3 was orally administered once daily for 11 days. Intracranial tumors were viewed using MRI. The body weights of mice were monitored once every 2 or 3 days. The feeding and handling of animals have been approved by the Animal Care & Welfare Committee Institute of Materia Medica, CAMS & PUMC (Approval numbers: 00008485, 00008487, 00008963).

### 2.22 Immunohistochemistry (IHC)

IHC was performed following standard protocols. Briefly, the standard protocol for Ki67 immunohistochemical staining involved tissue preparation, deparaffinization and rehydration, antigen retrieval, blocking, primary and secondary antibody incubations, visualization with DAB, counterstaining, mounting, microscopic examination, and image acquisition. To assess proliferation rates in tissue samples, the area of Ki67 expression was used for comparison in each group.

### 2.23 MRI analysis

After 11 days of treatment, anatomical images of the intracranial tumor were acquired using an MRI scanner (PharmaScan 70/16 US, Bruker, Switzerland) on the nude mice. The parameters used in the scans to optimize gray/white matter contrast were as follows: a T2_TurboRARE, with TR/TE = 2795/35, 4 averages, 22 × 22 field-of-view, and 0.5 mm slice thickness. The scanning of all brains was completed in 25 sessions. The tumor volume was calculated using the sum of the areas in all images multiplied by the thickness of each slice (0.5 mm).

### 2.24 Statistical analysis

All the statistical analyses and data fitting were conducted using GraphPad Prism version 8.0.2. Statistical significance was assessed by conducting a two-tailed Student's *t*-test or one-way ANOVA. The *P* values < 0.05, <0.01, or <0.001 were considered to indicate statistical significance, whereas “ns” denoted no statistical significance.

## 3 Results

### 3.1 PF403 exhibits potent antitumor activity

Phenanthroindolizidine alkaloids are a class of plant-derived small molecule compounds with a wide range of remarkable pharmacological activities, including potent antitumor activities^33^. CAT (Fig. 1A) was first obtained from the plants *Tylophora atrofolliculata* and *Tylophora ovata*. Compared to CAT, a metabolite of CAT called PF403 (Fig. 1A) demonstrated better antitumor activity (Fig. 1B). In animal models, PF403 was rapidly transformed to the glucose aldehydeylated metabolite GLU-PF403, which increased the polarity of PF403 and resulted in its rapid clearance^20^. Hence, CAT3 was designed and synthesized as a prodrug of PF403 and displayed significant *in vivo* efficacy against glioma growth (Fig. 1A)^17^.

Therefore, in subsequent investigations, PF403 was mainly used for molecular and cellular level experiments, while CAT3 (a prodrug of PF403), which exhibits improved pharmacokinetic properties, was used at the animal level. Moreover, conclusive evidence demonstrated that these three molecules could penetrate the blood–brain barrier and readily localize to brain tissue after administration^17^^,^^20^. The IC_50_ inhibition values of the three alkaloids against U87 cells were 23.4, 0.59, and 12.27 nmol/L, respectively (Fig. 1B). And PF403 displays significant suppression on several malignant tumor cell lines of MDA-MB-231, SK-MEL-5, MCF-7-T, SH-SY5Y, HepG2, and K562 with IC_50_ values of 6.24, 1.64, 3.42, 1.51, 2.54 and 2.47 nmol/L, respectively (Fig. 1C).

### 3.2 Experimental procedure for TPP and analysis results for ProSAP

Activity-based protein profiling (ABPP) is a classical chemical biology method used for target identification of active molecules^34^^,^^35^. However, in the case of PF403, the activity-holding probe P1 could not label the protein efficiently because the ester bond was likely broken at the cellular level. Although the ether-bonded probe can label intracellular proteins in a concentration-dependent manner, its antitumor inhibitory effect on SH-SY5Y cells was reduced by four orders of magnitude (Supporting Information Fig. S1). As expected, probes P3 and P4, which lack photocrosslinking groups and exhibited partially maintained activity, cannot effectively label proteins. We attempted cellular *in situ* labeling and target pull-down experiments with the activity-decreasing probe P2 to search for potential targets but failed in subsequent validation (Fig. S1).

Recently, a series of modification-free approaches for target identification, including TPP, have attracted widespread attention^12^^,^^28^^,^^36^, ^37^, ^38^. Therefore, we chose the alternative strategy (TPP) based on protein thermal stability to explore the direct target PF403. In general, proteins denature and precipitate with increasing heating temperature. However, thermal stability can be improved when proteins bind small molecules; compared to native proteins, these products are less prone to denaturation and precipitation at the same temperature.

**Figure 2:**
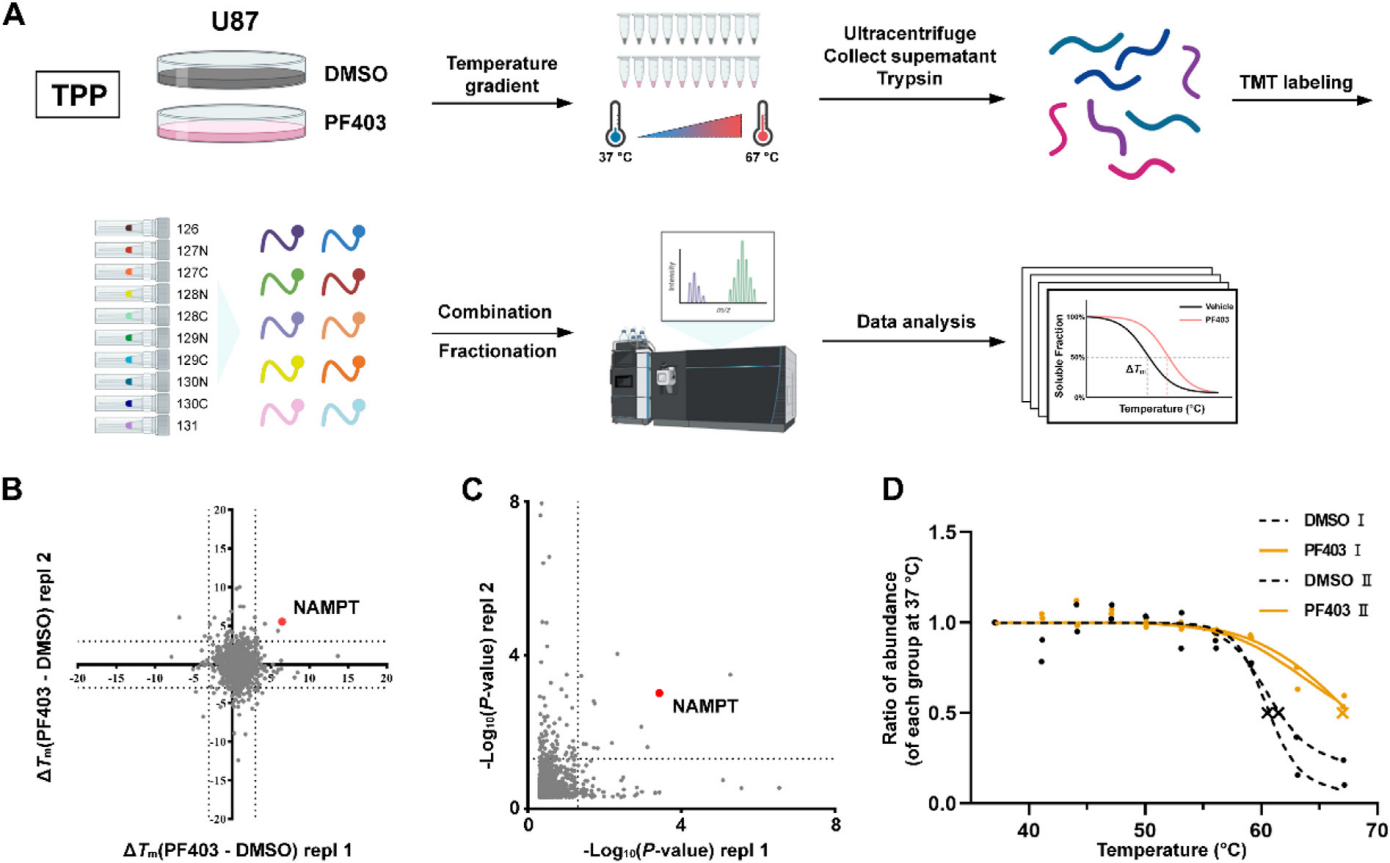
Nicotinamide phosphoribosyl transferase (NAMPT) is a cellular target of PF403 identified by thermal proteome profiling (TPP). (A) A workflow for identifying cellular targets of PF403 *via* TPP was created with BioRender.com. (B) Scatter plot of melting temperature (*T*_m_) shifts determined from two biological replicates of DMSO *versus* PF403 treatment. The data were processed using Proteome Discoverer (2.3), and ProSAP software was used to fit the thermal response curve and calculate the melting point. (C) Scatter plot of *P* values determined from two biological replicates of DMSO *versus* PF403 treatment. (D) Thermal response curves and calculated melting points for NAMPT in cells treated with PF403 (displayed in orange) and DMSO (displayed in grey). The graphs depict two biological duplicates.

The U87 cells were incubated with 10 μmol/L PF403 or DMSO for 3 h and then exposed to different temperatures for 3 min. The isolated proteins were labeled with tandem mass tag 10 (TMT10), subjected to UPLC fractionation, and analyzed by LC–MS/MS (Fig. 2A). After the mass spectrometry data was analyzed with a Proteome Discoverer (2.3) workstation, the temperature-content curves were fitted to the proteins of the two datasets using the R language package and ProSAP software.

There are a total of seven specific requirements for screening proteins in the R package (Supporting Information Table S1), but there were no proteins that met all requirements. As another option, the GUI software ProSAP was used to process the protein data, which resulted in four proteins meeting the specific requirements of ProSAP and obtaining scoring values^39^ (Supporting Information Table S2). Melting temperature (*T*_m_) shifts were calculated based on two replicates of PF403 compared to DMSO treatment and visualized after filtering (Fig. 2B). The *P*-value of the results was calculated by negative logarithms and is presented in a scatter plot (Fig. 2C).

When analyzing the melting curve of NAMPT fitted by TPP R package (Supporting Information Fig. S2), we found that the ratio of NAMPT content was still far greater than 50% under the maximum temperature of 67 °C. As a result, the fitted analyses by TPP R-package could not identify the *T*_m_ of the treatment group (two replicates). Especially for the P1 group, due to the limited range of curve descent, the slope value was not calculated. Due to the inability to calculate the melting point of the treatment group, the last three of the seven target screening criteria cannot be met. The parameters related to slope and *P*-value are also slightly beyond the required range. Therefore, NAMPT was excluded from this screening. The ProSAP software intelligently sets the melting point to 66.99 °C for proteins that still contain more than 50% at the highest temperature. Besides, in the output table, ProSAP sorts all the potential targets with a harmonized score combining both the significance of Δ*T*_m_ and the goodness-of-fit (*R*^2^).

Among the four proteins identified by ProSAP, SAP30BP is not closely related to the growth and development of cells based on an extensive literature review. Although the literature suggests that the two target genes (TP53BP1 and RPL8) and cancer development are associated, possible false positives may occur because the difference in temperature is too small. Therefore, temperature-dependent cellular thermal shift assays (CETSA), combined with Western blot (WB) experiments, were performed on RPL8 and TP53BP1 with three replicates for validation. However, there was no significant difference in temperature-dependent CETSA with the addition of PF403 (Supporting Information Fig. S3). These two proteins were therefore excluded. Based on this analysis, the three proteins were excluded as target targets. Notably, a temperature gradient of 5.99 °C between PF403 and DMSO was observed in the specific thermal stability curve of NAMPT identified by TPP (Fig. 2D). NAMPT has been reported to be the rate-limiting enzyme of NAD ^+^ biosynthesis and is overexpressed in several types of cancer cells to satisfy the continuous requirement for rapid proliferation^40^. Therefore, it was speculated that NAMPT is involved in the process of cell death caused by PF403.

### 3.3 NAMPT exhibits good binding affinity with PF403

**Figure 3:**
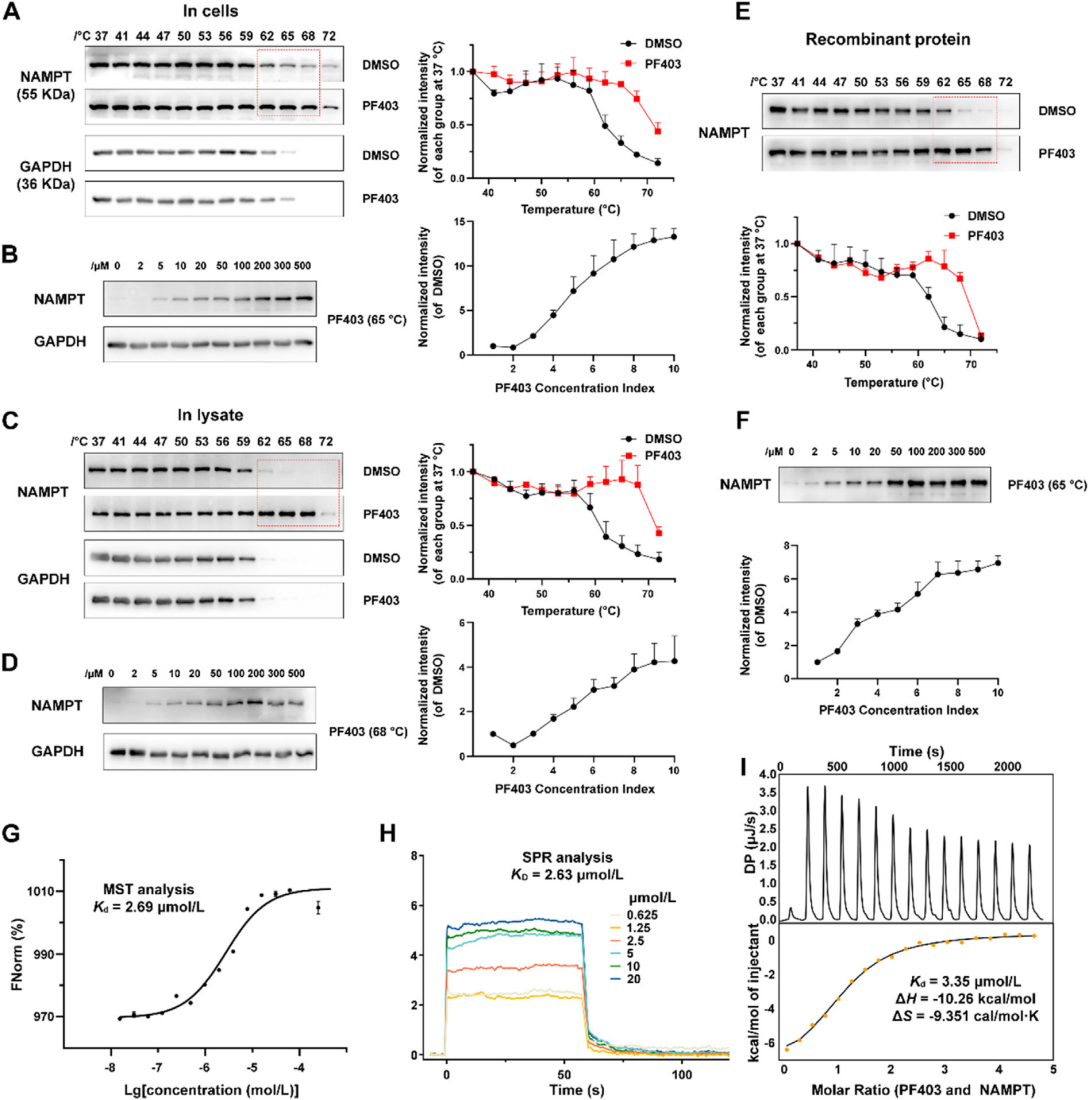
PF403 binds to NAMPT. (A) PF403 increases the thermal stability of NAMPT in living cells in a temperature-dependent manner, as determined by a cellular thermal shift assay (CETSA) (*n* = 3). (B) The thermal stability of NAMPT was measured using a dose-dependent CETSA at 65 °C (*n* = 3). (C, D) PF403 increases the thermal stability of NAMPT in cell lysates in a temperature- and concentration-dependent manner, as determined by CETSA (*n* = 3). (E, F) PF403 increases the thermal stability of recombinant NAMPT in a temperature- and concentration-dependent manner, as determined by CETSA (*n* = 3). (G) Microscale thermophoresis (MST) assay showing the binding affinity of PF403 for NAMPT (*n* = 3). (H) Surface plasmon resonance (SPR) analysis of the interactions between NAMPT and PF403. (I) Isothermal titration calorimetry (ITC) titration of PF403 with NAMPT. All data are expressed as the mean ± SD.

We validated the interaction between PF403 and NAMPT in U87 cells using CETSA^41^. Our results showed that PF403 significantly increased the stability of NAMPT. After fixation at 65 °C, the amount of soluble NAMPT increased with increasing concentrations of PF403 (Fig. 3A and B). In addition, temperature- and dose-dependent CETSAs of the cell lysates revealed that the thermal stability of NAMPT was affected by PF403 but not GAPDH (Fig. 3C and D). Consistently, PF403 caused a relatively large thermal shift in the recombinant form of NAMPT (Fig. 3E), and the change was concentration-dependent (Fig. 3F).

Moreover, the MST experiment demonstrated that PF403 is directly bound to NAMPT with a dissociation constant (*K*_d_) of 2.69 μmol/L (Fig. 3G). Furthermore, we quantitatively investigated the interaction between PF403 and NAMPT using SPR technology and obtained a *K*_D_ value of 2.63 μmol/L (Fig. 3H). The ITC assay was performed to measure the *K*_d_ value at 3.35 μmol/L (Fig. 3I). NanoDSF was carried out to monitor the changes in endogenous fluorescence signals of proteins during thermal shifts and to calculate their *T*_m_ values. The results of the nanoDSF experiments showed that PF403 increased the thermal stability of NAMPT, indirectly confirming that PF403 can bind to NAMPT (Supporting Information Fig. S4).

### 3.4 The pi–pi interaction between PF403 and Y188 is essential for NAMPT–PF403 binding

**Figure 4:**
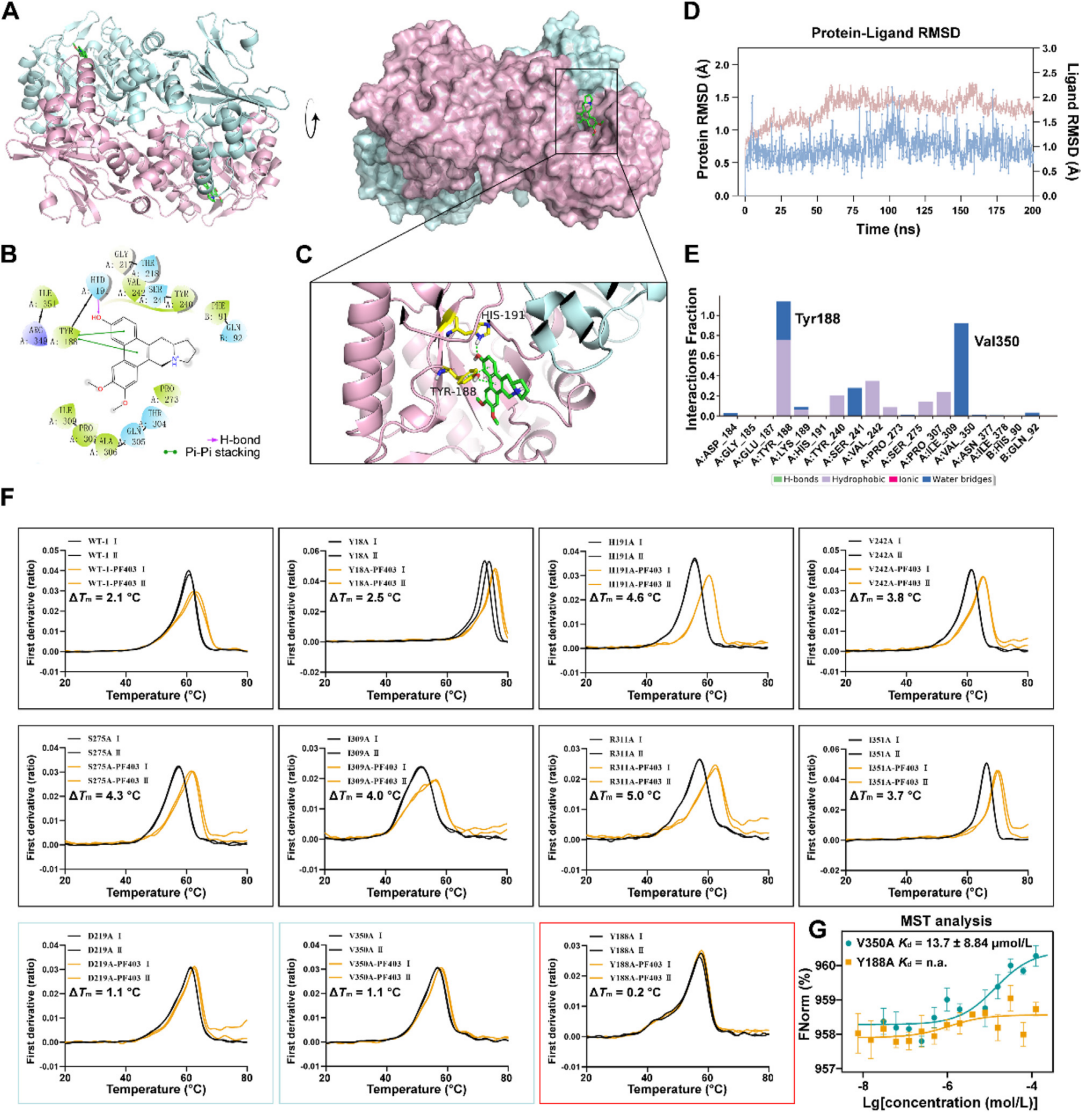
Cocrystal structure of NAMPT (dimer) in complex with PF403 (PDB code 8Y55). (A) The binding surface of PF403 (green) with the NAMPT catalytic pocket. (B) Ligand–protein interaction between PF403 and NAMPT (2D diagram). (C) Details of the NAMPT–PF403 complex imaged in 3D. (D) Root mean square deviation (RMSD) values (Å) of NAMPT and PF403 in the 200 ns MD simulation. (E) Bar charts of protein–ligand interactions obtained from MD simulation. (F) Alanine mutants of ten key amino acids around the NAMPT active pocket were expressed and purified, after which the wild-type (WT) and mutated NAMPT strains treated with PF403 (displayed in orange) and DMSO (displayed in grey) were subjected to nanodiometric scanning fluorescence (nanoDSF) assays. (G) PF403 lost its binding affinity for Y188A NAMPT, as determined by MST analysis (*n* = 3). All data are expressed as the mean ± SD.

Subsequently, cocrystal culture was performed with recombinant NAMPT and PF403. Fortunately, a 1.86 Å cocrystal was successfully grown, and single X-ray diffraction data were collected (Supporting Information Table S3). NAMPT is a natural dimer in its physiological state that contains two PF403 molecules bound in its active pocket (Fig. 4A). PF403 was embedded in the active cavity of NAMPT, as represented by the protein surface model (Fig. 4A). The interaction patterns of the NAMPT–PF403 cocrystal complex were assessed using Maestro (Schrödinger software package), and the results revealed that residue His191 formed hydrogen bonds with the phenolic hydroxyl group of PF403 and that residue Tyr188 formed pi–pi interactions with the large pi-bonds of PF403 (Fig. 4B and C).

Molecular dynamics simulation is a significant method for studying the stability of complex postures and the contributions of key amino acids in a simulated physiological environment^42^. We performed MD simulations with NAMPT and PF403 for 200 ns by using the Desmond module. The parameters of the NAMPT–PF403 complex were monitored during the simulation to detect changes in residue conformations within the active site. The root mean square deviation (RMSD) values of the protein backbone atoms were calculated to estimate the stability of the protein and ligand (Fig. 4D). The RMSD of the NAMPT state gradually reached a steady value of approximately 1.4 Å after 60 ns. Protein–ligand interactions involving hydrogen bonding and hydrophobic, ionic, and water bridges were monitored throughout the entire simulation and are shown as a histogram according to the percentage of duration (Fig. 4E). After simulation, crucial contacts with Tyr188 (76% hydrophobic contacts and 38% water bridges) were found to be maintained, consistent with the previous interaction analysis by Maestro. A value of 76% indicated that a specific interaction occurred during 76% of the entire simulation time. Instead of hydrophobic contacts relative to residue Tyr188, several other hydrophobic interacting amino acids appeared during the simulation, *e.g.*, Tyr240 (20%), Val242 (35%), and Ile309 (24%). Notably, residue Val350 reached 92% contact with proteins through water bridges. The simulation interaction diagram showed that hydrophobic and water–bridge interactions played important roles in the binding affinity of the NAMPT–PF403 complex within the active site (Fig. 4E).

Subsequently, we performed an alanine scan on the NAMPT–PF403 complex (Supporting Information Fig. S5). Combining the analysis of alanine scanning results and molecular dynamic results, ten amino acids were found to be mutated, including Y18A, Y188A, H191A, D219A, V242A, S275A, I309A, R311A, V350A, and I351A. The gene encoding the mutation was inserted into the *E. coli* expression vector, after which the mutated proteins were subsequently expressed and purified. NanoDSF experiments were also conducted on the ten mutant and wild-type (WT) proteins, which indicated that nine of the mutant proteins were significantly more thermally stable (Fig. 4F). The thermal stability of the Y188A mutant protein was not improved when PF403 was added. Notably, the D219A and V350A NAMPTs exhibited a slight increase of only 1.1 °C in thermal stability when PF403 was added, suggesting that certain interactions occurred. Residue Val350 was also demonstrated to form a strong water bridge with PF403 *via* molecular dynamics simulation. However, in the subsequent MST experiments, the V350A mutation resulted in a reduced loss of binding affinity with PF403 (Fig. 4G). Only the Y188A mutant completely lost its ability to bind PF403, as identified by MST (Fig. 4G). These results demonstrated that residue Tyr188 was the key to maintaining NAMPT–PF403 binding, which was consistent with the MD simulation results.

(*E*)-Daporinad (FK866) is an effective inhibitor of NAMPT with a reported *K*_i_ of 0.3 nmol/L^43^. The binding positions of PF403 and FK866 in NAMPT were superimposed to visualize the difference between the two types of inhibitors (Supporting Information Fig. S6). FK866 occupies the catalytic pocket of the original substrate nicotinamide mainly in a linear pose, whereas PF403 blocks the entrance to the active pocket like a “goalkeeper”. PF403 and FK866 are two completely different binding modes, and we believe that a stronger NAMPT inhibitor might be produced if the two inhibitors are spliced together in a suitable mode. This approach would be very meaningful and worth developing.

### 3.5 PF403 inhibits the catalytic activity of NAMPT

**Figure 5:**
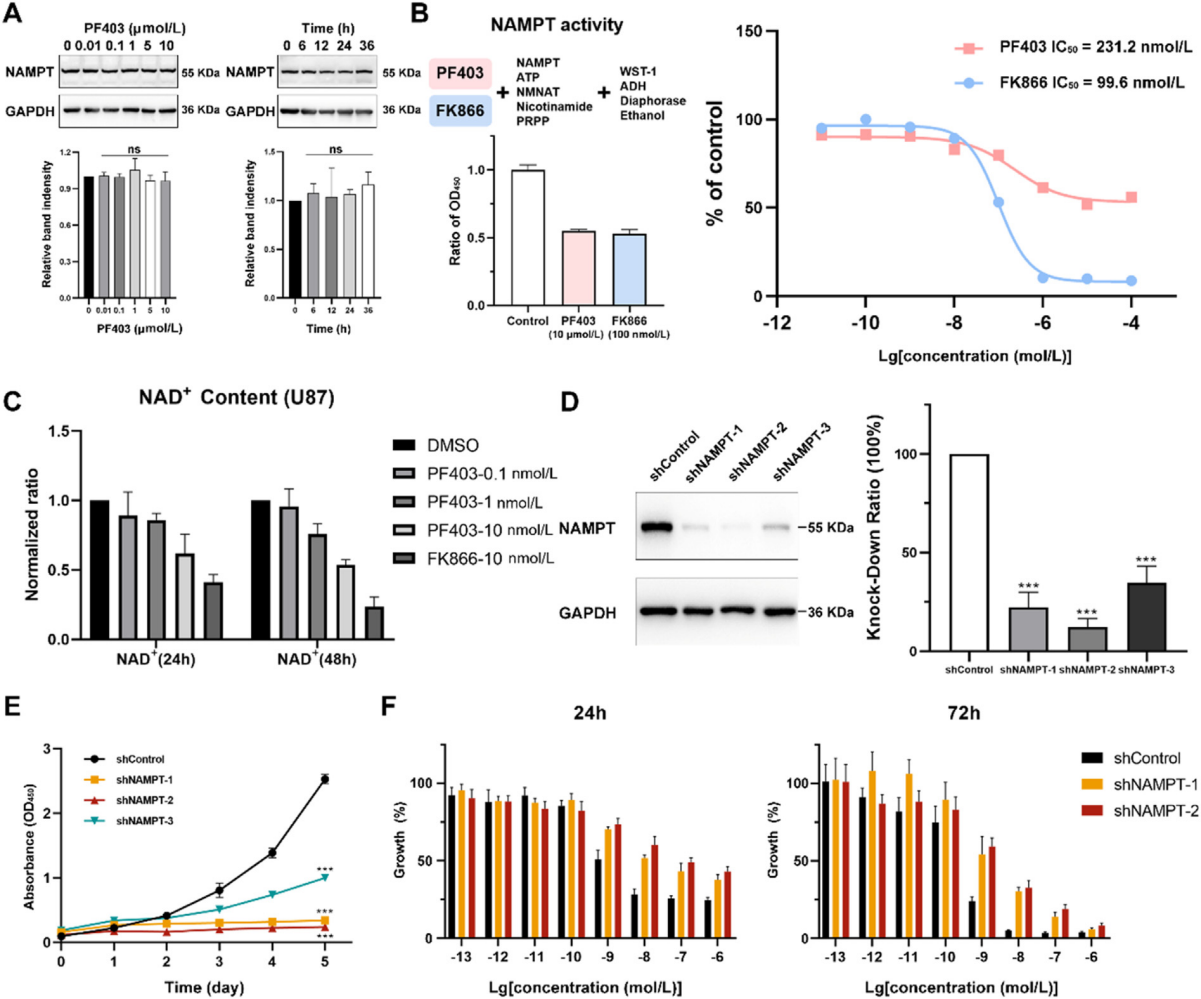
PF403 inhibits the activity of NAMPT rather than reducing its concentration. (A) PF403 did not change the intracellular content of NAMPT at different times (*n* = 2) or concentrations (*n* = 4). (B) The indirect inhibitory effects of PF403 (10 μmol/L) and FK866 (100 nmol/L) on NAMPT activity. (C) The Nicotinamide adenine dinucleotide (NAD^+^) concentration in U87 cells was detected after PF403 treatment for 24 and 48 h (*n* = 3). (D) Knockdown of NAMPT in U87 cells *via* lentiviral transduction *via* three interfering chains. NAMPT knockdown was detected *via* Western blotting (WB) (*n* = 4). (E) Growth curves of wild-type U87 cells and three types of NAMPT-knockdown U87 cells (shControl, shNAMPT-1, shNAMPT-2, and shNAMPT-3) (*n* = 5). (F) U87 cells with NAMPT knockdown were treated with vehicle or PF403 at the indicated concentrations for 24 or 72 h (*n* = 5). All data are presented as the mean ± SD. Statistical significance was assessed by one-way ANOVA test, ∗*P* < 0.05, ∗∗*P* < 0.01, and ∗∗∗*P* < 0.001.

Furthermore, we investigated how binding to NAMPT affects the biological function of PF403. First, we examined the effect of PF403 on the expression of NAMPT at different times and different concentrations. Clearly, PF403 did not cause changes in the levels of NAMPT in cells (Fig. 5A). The indirect activity of NAMPT was also analyzed using a NAMPT activity kit. PF403 and FK866 significantly inhibited the activity of NAMPT (Fig. 5B). Due to the poor solubility of PF403, the inhibition curve of NAMPT activity did not increase as the concentration increased. However, we observed that at 10 μmol/L, PF403 inhibited the enzymatic activity of NAMPT by more than 50%.

NAMPT can catalyze the transformation of NAM to add a ribose phosphate group to NMN (Supporting Information Fig. S7)^44^. We tested the direct enzyme activity of PF403. Unfortunately, PF403 exhibits background fluorescence. The inhibitory effect of PF403 on NAMPT activity was determined by background subtraction (Fig. S7). There was a clear tendency to inhibit enzyme activity, although the results were not consistent with the standard “S” type. In addition, we determined the amount of NAD^+^ in living cells after PF403 was added^45^^,^^46^. The content of intracellular NAD^+^ was inhibited in a dose-dependent manner after 24 and 48 h (Fig. 5C). At a concentration of 10 nmol/L, the positive drug FK866 has a better inhibitory effect on intracellular NAD^+^ content than PF403.

To confirm that NAMPT is a functional target of PF403, we knocked down the NAMPT gene in the U87 cancer cell line with three shRNA interference sequences by lentiviral infection. After these cells were selected using puromycin, stable NAMPT gene-knockdown cell lines (shNAMPT-1, shNAMPT-2, and shNAMPT-3) were constructed. After stably transfected cell lines were selected, WB was performed to detect the expression of NAMPT in different shRNA-transfected cell lines (Fig. 5D). The results indicated that the expression of NAMPT was markedly suppressed by shRNA. The shNAMPT-1 and shNAMPT-2 cell lines were knocked down more effectively, with 22.4% and 12.3% of the target protein remaining, respectively (Fig. 5D).

Next, we determined the growth curves of the shControl and the three knockdown cell lines by using a CCK-8 kit. The results showed that the knockdown of NAMPT in U87 cells led to slow and even arrested growth (Fig. 5E). We observed that the shNAMPT-1 and shNAMPT-2 cell lines still grew, and both cell lines slowly gained the ability to grow in continuous culture despite continuous screening with puromycin.

To determine whether the inhibitory effect of PF403 on tumor cells was decreased *via* NAMPT knockdown, we measured the anti-proliferative effect of PF403 on transfected U87 cells after 24 and 72 h. After adding PF403 for 24 h, we found that PF403 exhibited no significant anti-proliferative effect on the WT, shNAMPT-1, or shNAMPT-2 cell lines at concentrations between 10^−13^ and 10^−10^ mol/L. However, when the concentration of PF403 was 10^−9^ and 10^−6^ mol/L, the toxicity of PF403 to the WT cells increased rapidly, while the toxicity to the two knockdown cell lines increased slowly. At specific concentrations, differences were observed between the knockdown cells and WT cells, especially for the shNAMPT-2 cell line, which exhibited a better knockdown effect. After 72 h, we also observed similar results. Compared to that of the wild-type, the anti-proliferative effect of PF403 on the NAMPT stable-knockdown cell line was partially abolished at concentrations of 10^−9^ and 10^−6^ mol/L (Fig. 5F). The decreased anti-proliferative activity in stable NAMPT knockdown cells under PF403 demonstrated that NAMPT was an important functional target of PF403.

### 3.6 Effect of CAT3 (a prodrug of PF403) on the proliferation of U87 xenograft model (WT and NAMPT-knockdown cells)

**Figure 6:**
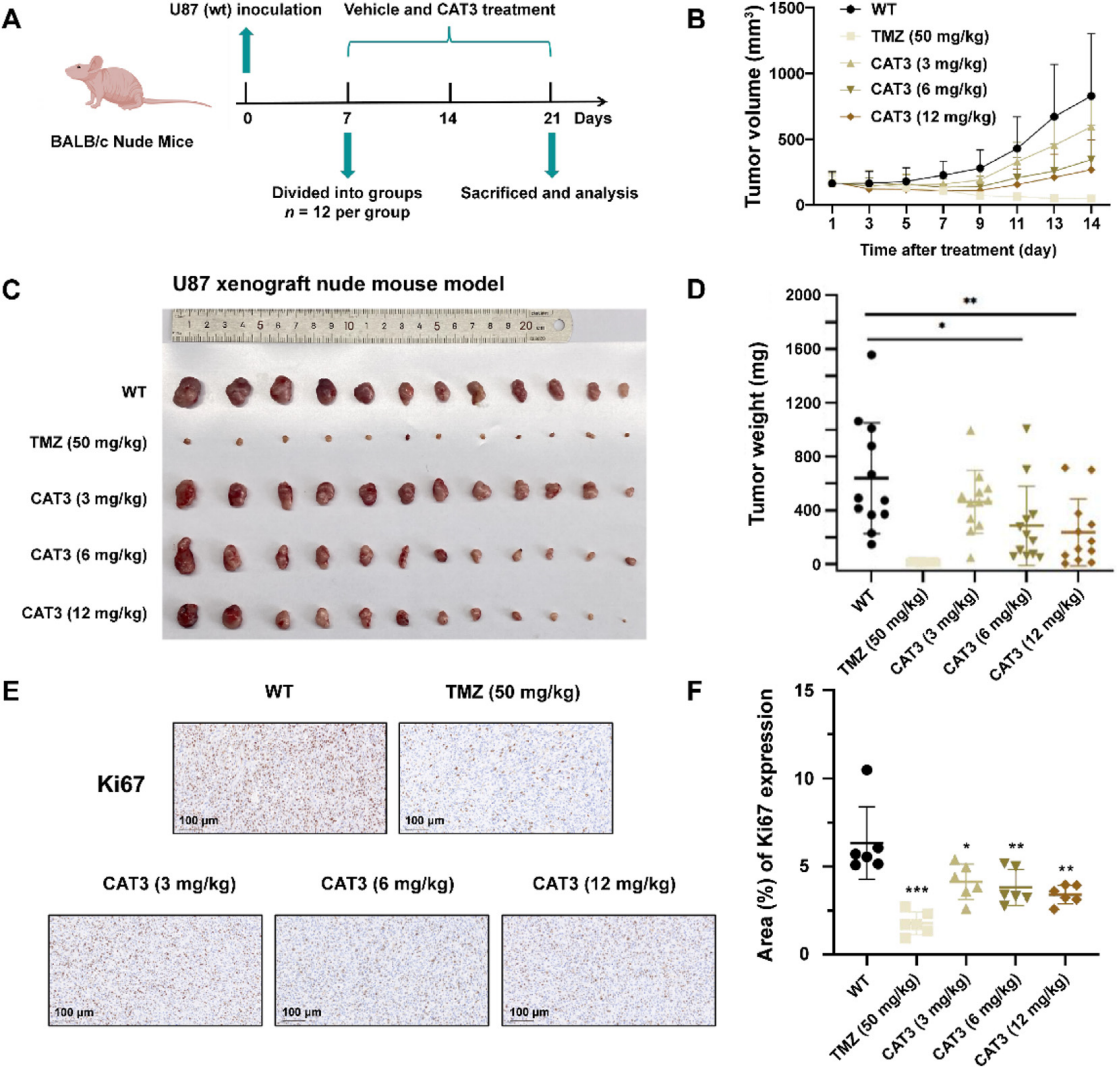
CAT3 (a prodrug of PF403) inhibits wild-type glioma tumor growth. (A) Schematic representation of the CAT3 efficiency experimental protocol (created with BioRender.com, *n* = 12). (B) Xenograft tumor volume curve for 1–14 days. CAT3 dose-dependently inhibited tumor growth in the U87 xenograft model (i.g., daily for 14 days, *n* = 12). (C) Images of tumors were dissected from each mouse on the day the mice were sacrificed (*n* = 12). (D) Tumor weights in different groups on the day the mice were sacrificed (*n* = 12). (E) Representative Ki67 IHC staining of tumor specimens (scale bar, 100 μm). (F) Quantification of Ki67 expression (% area) in tumor cells of different groups of mice (*n* = 6). All data are presented as the mean ± SD. Statistical significance was assessed by one-way ANOVA test, ∗*P* < 0.05, ∗∗*P* < 0.01, and ∗∗∗*P* < 0.001.

To evaluate the effect of PF403 on the proliferation of U87 cells *in vivo*, we established a xenograft model of U87 cells (Fig. 6A). The growth of tumor was markedly suppressed in the PF403 prodrug group compared to the model group. Based on the tumor volume data, the inhibitory effects of 6 mg/kg and 12 mg/kg CAT3 (a prodrug of PF403) were close to 58.41% and 67.59%, respectively (Fig. 6B and C). In terms of tumor weight on the 14th day, 6 and 12 mg/kg CAT3 (a prodrug of PF403) also achieved 55.37% and 63.18% inhibitory effects, respectively (Fig. 6D). The expression of Ki67 was reduced after treatment with CAT3 (a prodrug of PF403) (Fig. 6E and F), suggesting that PF403 inhibited the proliferation of U87 cells *in vivo*. Due to the gastrointestinal reaction induced by CAT3 (a prodrug of PF403), the body weight of the nude mice decreased slightly in the first week and then gradually recovered (Supporting Information Fig. S8).

**Figure 7:**
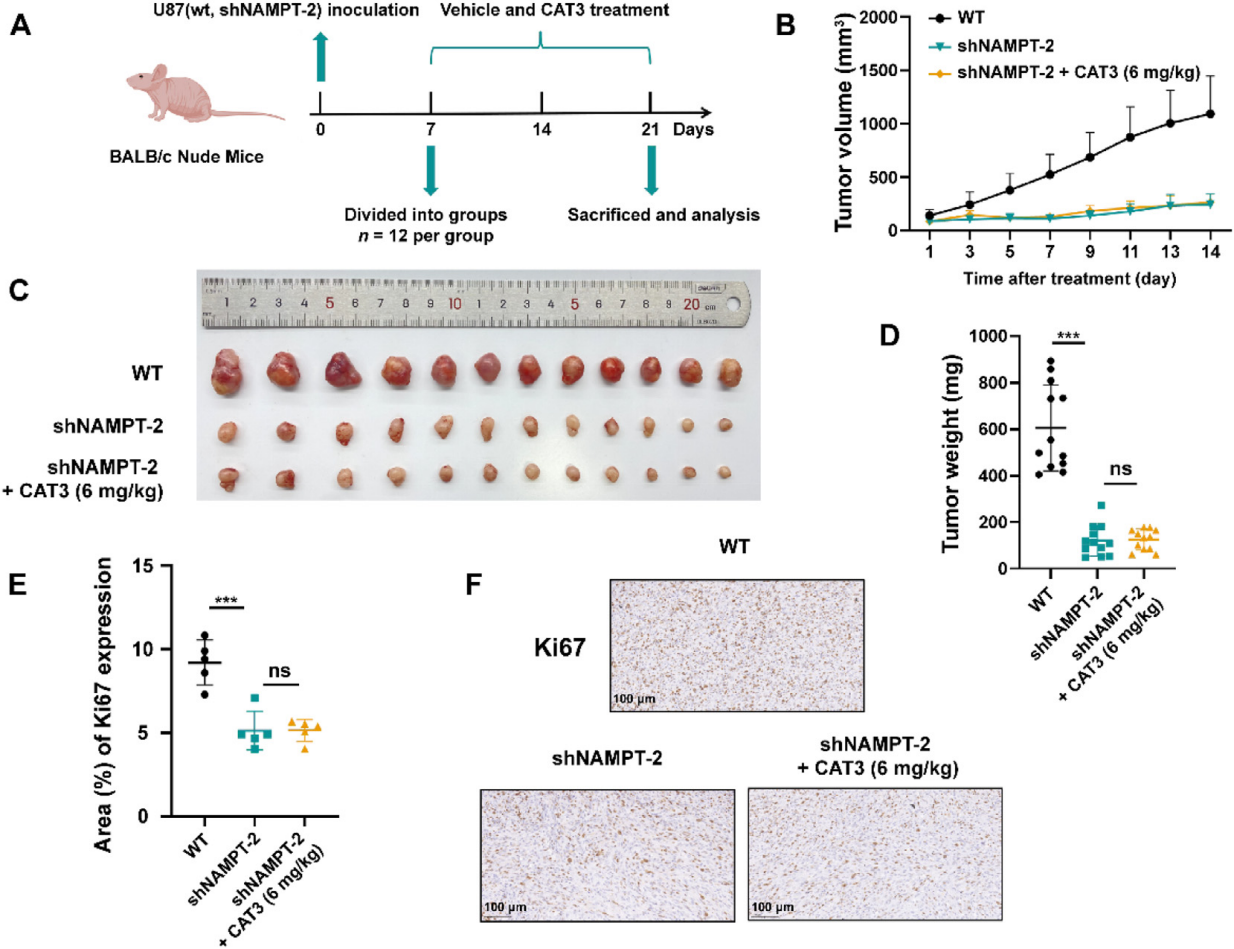
CAT3 (a prodrug of PF403) lost efficacy in NAMPT-knockdown tumors. (A) Experimental protocol of the tumor (NAMPT-knockdown cells) inhibitory effect by CAT3 (created with BioRender.com, *n* = 12). (B) Growth curve of wild-type and NAMPT-knockdown xenografts; one group was treated with an effective dose of CAT3 (6 mg/kg, i.g. daily for 14 days, *n* = 12). (C) Images of tumors were dissected from each mouse on the day the mice were sacrificed (*n* = 12). (D) Tumor weights in different groups on the day the mice were sacrificed (*n* = 12). (E) Quantification of Ki67 expression (% area) in tumor cells of different groups of mice (*n* = 5). (F) Representative Ki67 IHC staining of tumor specimens (scale bar, 100 μm). All data are presented as the mean ± SD. Statistical significance was assessed *via* two-tailed unpaired Student's *t*-test, ∗*P* < 0.05, ∗∗*P* < 0.01, ∗∗∗*P* < 0.001, and ns: no significance.

To further investigate the inhibitory effect of PF403 on stable-knockdown U87 cells, we established a xenograft model of stable-knockdown U87 cells (Fig. 7A). NAMPT deficiency significantly inhibited the proliferation of U87 cells in the xenograft model (Fig. 7B and C). Notably, CAT3 (a prodrug of PF403) had no significant inhibitory effect on cell proliferation in the xenograft model of shNAMPT-2 U87 cells, as determined by tumor volume or weight (Fig. 7B–D). Body weight was recorded over 14 days; similarly, a slight decrease in body weight was found in the prodrug PF403 (CAT3) treatment group due to gastrointestinal side effects (Supporting Information Fig. S9). Moreover, the expression of Ki67 was significantly reduced in the xenograft model of NAMPT-knockdown cells and was not affected by the CAT3 at effective dose concentration (Fig. 7E and F). The results at the animal level further confirmed that NAMPT is a key functional target of PF403.

**Figure 8:**
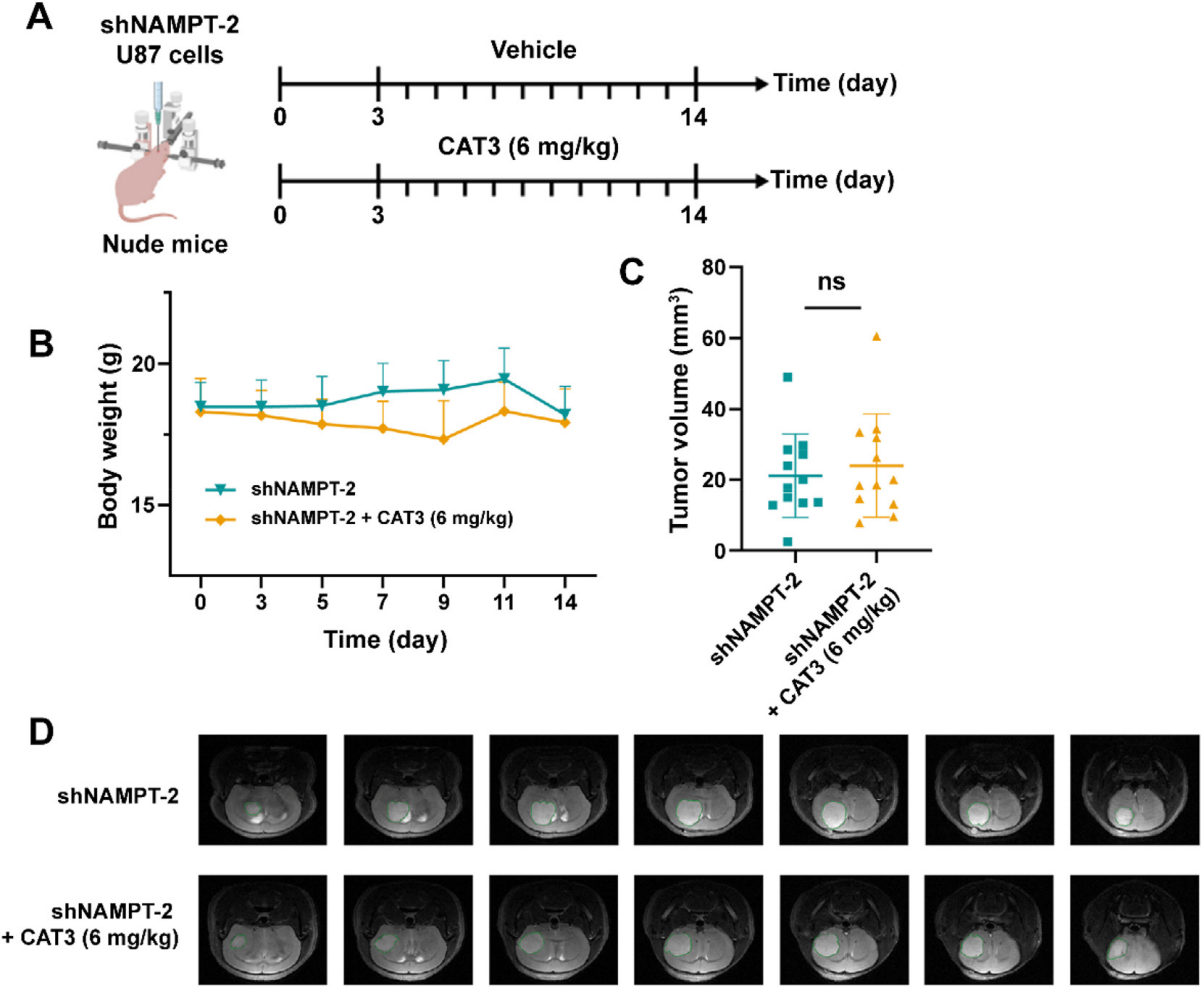
CAT3 (a prodrug of PF403) lost efficacy in the orthotopic glioma model with NAMPT knockdown cells. (A) Schematic of orthotopic xenograft model (created with BioRender.com). Treatment started on the third day after the injection of tumor cells and continued for 11 days (*n* = 12). (B) The body weights of the mice were monitored for 14 days (*n* = 12). (C) Comparison of orthotopic glioma volumes among different groups on the 11th day after CAT3 treatments (*n* = 12). (D)The T2-weighted enhanced MRI images of the tumor on the 14th day (representative images). All data are presented as the mean ± SD. Statistical significance was assessed *via* two-tailed unpaired Student's *t*-test, ns: no significance.

In our previous study, anti-tumor activity was evaluated in an orthotopic glioma model with U87 cells^17^. The results showed that 6 mg/kg of CAT3 had a near 40% inhibitory effect on the U87 cell line during 11 days of continuous treatment. Therefore, to validate the function of PF403 in the NAMPT knockdown orthotopic glioma model, we established the model with shNAMPT-2 U87 cells and used the effective dose (6 mg/kg CAT3) for a treatment lasting 11 days (Fig. 8A). The body weight of the nude mice was monitored every 2 or 3 days (Fig. 8B) and T2-weighted enhanced MRI images of the tumor was obtained on the last day (Fig. 8D). The tumor volume of each group of 12 mice was calculated using RadiAnt DICOM software by multiplying the total area of each piece by the thickness of 0.5 mm (Fig. 8C and Supporting Information Fig. S10). There was a slight decrease in the body weight of nude mice in the early and middle stages of the treatment group but no significant difference in final tumor volume. The treatment by PF403 did not result in a reduction of NAMPT knockdown tumor volume, which was 21.16 ± 11.28 and 24.05 ± 13.99 mm^3^ in the vehicle group and treatment group, respectively. The results from animal studies in the orthotopic glioma model further confirm that NAMPT is an important functional target of PF403 to exert anti-tumor functions *in vivo*.

## 4 Discussion

Applications of natural products are limited because research on the underlying mechanisms is lacking. Many natural products are structurally intricate and unsuitable or difficult to modify, and chemical modifications may affect target-molecule binding. Therefore, identifying target proteins with chemically unmodified small molecules, including DARTS (drug affinity responsive target stability), SPROX (stability of proteins from rates of oxidation), and TPP, is crucial. Through the TPP method, changes in protein thermal stability across the proteome can be monitored using quantitative mass spectrometry. In recent years, due to the high efficiency and convenience of TPP, this method has been increasingly used in the identification of natural products *via* molecular targeting. For instance, by applying TPP, researchers confirmed that lycorine directly targets a unique C-terminal domain of IDH1 to induce an imbalance in ROS-mediated mitochondrial dynamics^38^. In another study combining ABPP and TPP, LDC7559 was identified to target the PFKL (phosphofructokinase-1 liver type) to inhibit the NOX2-dependent oxidative burst in neutrophils^37^.

NAMPT affects tumor cell proliferation and differentiation by participating in the NAD^+^ salvage pathway. As an important cofactor, NAD^+^ is involved in numerous life processes, including cellular energy metabolism, DNA transcription and repair, and redox homeostasis. Compared to normal cells, tumor cells require more NAD^+^ due to their high energy demand. In mammals, NAD^+^ synthesis is largely dependent on the nicotinamide salvage pathway, in which NAMPT is the rate-limiting enzyme. Therefore, NAMPT is closely associated with tumor development. Although several NAMPT inhibitors (FK866, CHS-828, and EB-1627) have been introduced into the clinic, they ultimately failed in phase II clinical trials due to dose-limiting toxicities, including thrombocytopenia and retinal and cardiac toxicity^47^. Hence, to enhance the targeting ability of the NAMPT inhibitor, Bayer AG and Seagen designed and synthesized NAMPTi-ADCs for cancer therapy^48^^,^^49^. NAMPT inhibitors associated with DNA-alkylating agents were also created as a new strategy for inducing catastrophic NAD^+^ depletion by concurrently impairing NAD^+^ synthesis and promoting NAD^+^ consumption^50^. The poor efficacy of NAMPT inhibitors may result from the nonenzymatic activity of NAMPT and the cancer-promoting activity of extracellular NAMPT (eNAMPT), which is a cytokine-like protein. Therefore, the PROTAC molecule of NAMPT was designed to degrade intracellular NAMPT (iNAMPT) and eNAMPT^40^^,^^51^^,^^52^. Although these new development strategies have achieved impressive results in animal models, they remain in the preclinical research phase and need to be further validated. Thus, a novel structure of the NAMPT inhibitor has yet to be developed.

In this TPP experiment, we determined U87 intracellular thermal profiles for up to 4000 proteins and compared the differences in the presence or absence of PF403. In addition, the thermal stability of NAMPT significantly changed in the presence of PF403. Subsequently, the strong physical binding of NAMPT to PF403 was confirmed by MST, SPR, nanoDSF, temperature- and concentration-dependent CETSA experiments. In subsequent direct enzyme activity, indirect enzyme activity, and intracellular NAD^+^ content assays, PF403 inhibited the catalytic activity of NAMPT. Fortunately, we cultured and obtained a cocrystal of PF403 that is bound to NAMPT and found that PF403 binds to the rear channel of NAMPT active site. Perhaps the investigation of the non-enzymatic effects of PF403 on eNAMPT deserves further attention. To explore the specific binding mode of the surrounding amino acids, we also performed an alanine scan and molecular dynamics simulation to identify potential key residues. These potential key amino acids were subsequently mutated, expressed, and purified through an *E. coli* system, followed by nanoDSF experiments. Finally, the pi–pi contacts and water–bridge interactions formed by Tyr188 were the keys to maintaining the binding of PF403 to NAMPT. After NAMPT expression was subsequently knocked down, PF403 loses or departmentally loses its own antitumor activity at the cellular and animal levels due to the deletion of the NAMPT target. These findings further confirmed that NAMPT is a direct functional target of PF403.

Although our study verified that PF403 is a direct target of NAMPT, we cannot completely exclude the role of other proteins. Through other mass spectrometry data, we also showed that heme-binding protein 1 (HEBP1) binds PF403 by TPP and proteome integral solubility alteration (PISA) assays. Unfortunately, PF403 exhibited a partial loss of antitumor activity in the HEBP1 knockdown animal model, but this effect was not completely reversed. In addition to HEBP1, we speculated that the conjugated macrostructure of PF403 could also inhibit topoisomerase I and topoisomerase II through online target prediction and structure analysis. These findings were corroborated by subsequent enzyme activity tests, which showed the inhibitory effects at 38.34 and 87.33 μmol/L, respectively (Supporting Information Fig. S11). In previously published research by a collaborating group, we also demonstrated that it can bind to DNA and RNA^14^^,^^15^. This combination is also suspected to be the reason for partial activity and certain toxic side effects. Therefore, we would like to find the functional target for its direct binding at the level of the protein, enhance its targeting, and reduce toxicity through later modifications. Further exploration of the mechanism behind multi-target interactions is worthwhile in future studies.

## 5 Conclusions

Our research has identified a promising lead as an NAMPT binding agent for treating gliomas and revealed the precise interaction mode of the NAMPT–PF403 complex by X-ray diffraction. In summary, our findings reveal that PF403 could serve as a promising lead compound for treating cancer with high NAMPT expression, as NAMPT may play significant regulatory roles in multiple tumor types.

## Data availability

All data generated or analyzed during this study are included in this article and its Supporting Information files. More details are available from the corresponding authors upon request. Structures of the NAMPT–PF403 complex established by X-ray diffraction and accompanying data have been deposited in the Protein Data Bank (PDB, www.rcsb.org). Data and reagents requests should be addressed to corresponding authors.

## Author contributions

Fangfei Li: Formal analysis, Investigation, Data Curation, Writing - Original Draft, Visualization. Zhaoxin Zhang: Formal analysis, Investigation, Data Curation, Writing - Original Draft, Visualization. Qinyan Shi: Formal analysis, Investigation, Writing - Original Draft, Visualization. Rubing Wang: Investigation, Formal analysis. Ming Ji: Investigation, Formal analysis, Data Curation. Xiaoguang Chen: Conceptualization, Methodology. Yong Li: Methodology, Formal analysis, Data curation, Writing - review & editing, Supervision. Yunbao Liu: Methodology, Formal analysis, Data curation, Writing - review & editing, Project administration. Shishan Yu: Conceptualization, Methodology, Resources, Writing - Review & Editing, Supervision, Project administration, Funding acquisition.

## Conflicts of interest

The authors declare no competing financial interests.

## Appendix A Supporting information

The following is the Supporting Information to this article:
