# Supplementary material for: Thermal proteome profiling (TPP) reveals NAMPT as the anti-glioma target of phenanthroindolizidine alkaloid PF403
Source: Acta Pharm Sin B. 2025 Feb 26;15(4):2008–23. doi: 10.1016/j.apsb.2025.02.027 (PMC12138128; doi:10.1016/j.apsb.2025.02.027)
Supplement: Multimedia component 1 [file mmc1.pdf]

Supporting Information for

Original article

Thermal proteome profiling (TPP) reveals NAMPT as the anti-glioma target of phenanthroindolizidine alkaloid PF403

Fangfei Li†, Zhaoxin Zhang†, Qinyan Shi, Rubing Wang, Ming Ji, Xiaoguang Chen, Yong Li*, Yunbao Liu*, Shishan Yu*

State Key Laboratory of Bioactive Substance and Function of Natural Medicines, Institute of Materia Medica, Chinese Academy of Medical Sciences & Peking Union Medical College, Beijing 100050, China

Received 11 August 2024; received in revised form 20 October 2024; accepted 6 November 2024

†These authors made equal contributions to this work.

*Corresponding authors.

E-mail addresses: liyong@imm.ac.cn (Yong Li), liuyunbao@imm.ac.cn (Yunbao Liu), yushishan@imm.ac.cn (Shishan Yu).

Supporting Figures..2

Supporting Tables..12

Glossary..14

Supporting Figures

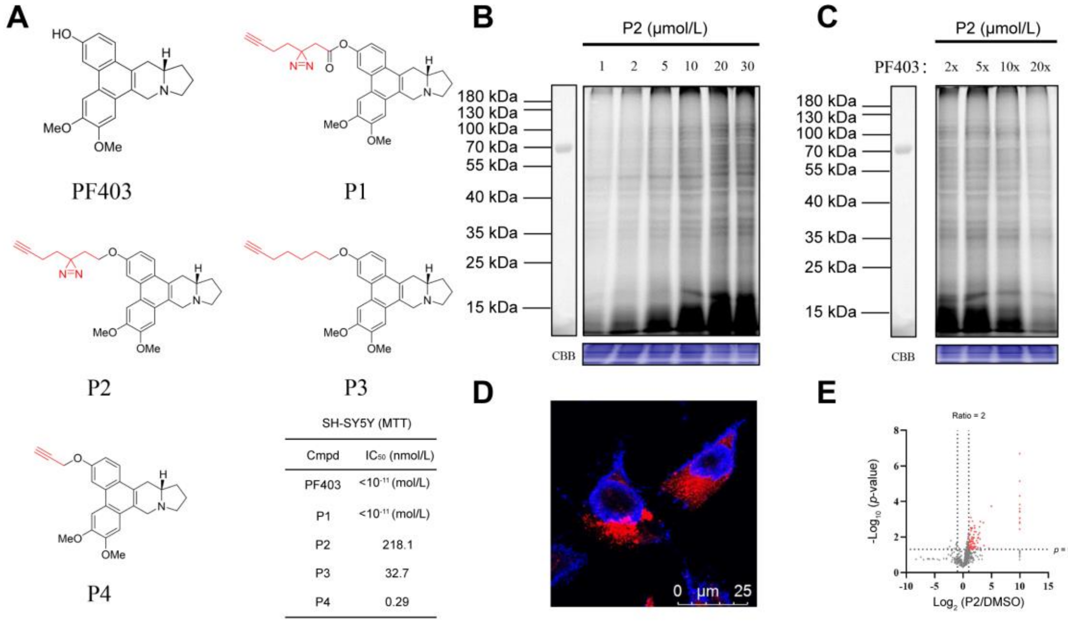

Figure S1 Identification of PF403 targets through the ABPP. (A) Chemical structures and the SH-SY5Y proliferation inhibition (72 h) of designed probes (P1, P2, P3 and P4). The IC_{50} units for PF403 and P1 are mol/L. (B) Concentration-dependent labeling with P2 at varied concentrations for 3 h in SH-SH5Y cells. (C) Competitive labeling of potential targets by P2 (10 mmol/L) and excessive PF403 (2-, 5-, 10- or 20- fold). (D) Live cell imaging of SH-SY5Y cells with P2 (10 mmol/L, 20 min UV irradiation), followed by cell fixation, permeabilization, click chemistry with TAMRA-N3, then image acquisition. Blue: Hoechst nuclear stain; red: TAMRA channel. (E) Volcano plot of enriched proteins in a competition experiment using the pull-down method with P2 (10 mmol/L)/DMSO (0.1%).

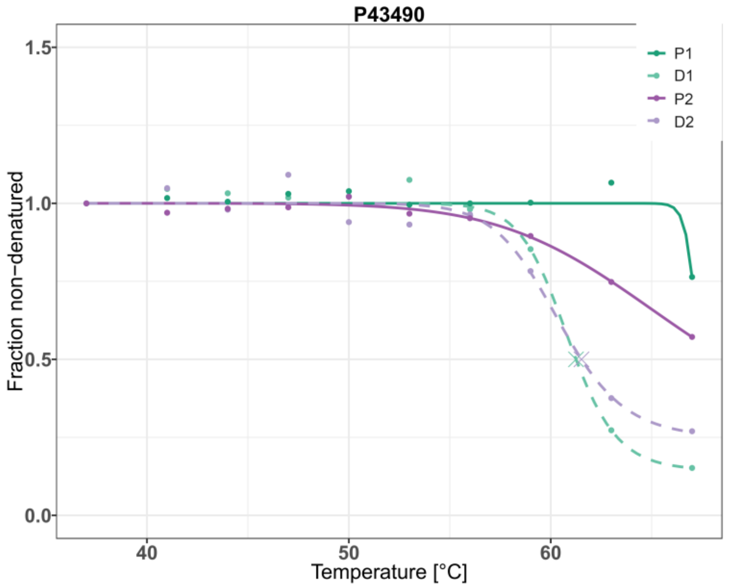

	meltPoint	slope	plateau	R2
P1	–	–	0	0.89
D1	61.25	−0.18	0.14	0.99
P2	–	−0.045	0.22	0.99
D2	61.52	−0.12	0.25	0.97

Figure S2 Specific parameters of the NAMPT melting curve fitted by the TPP R package.

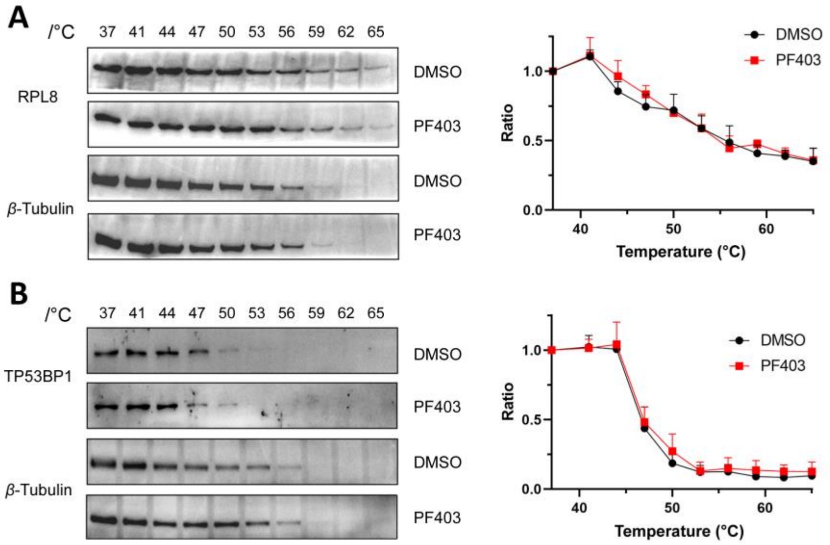

Figure S3 (A) Temperature-dependent CETSA of RPL8 in living cells (n = 3). (B)Temperature-dependent CETSA of TP53BP1 in living cells (n = 3). All data are expressed as the mean ± SD.

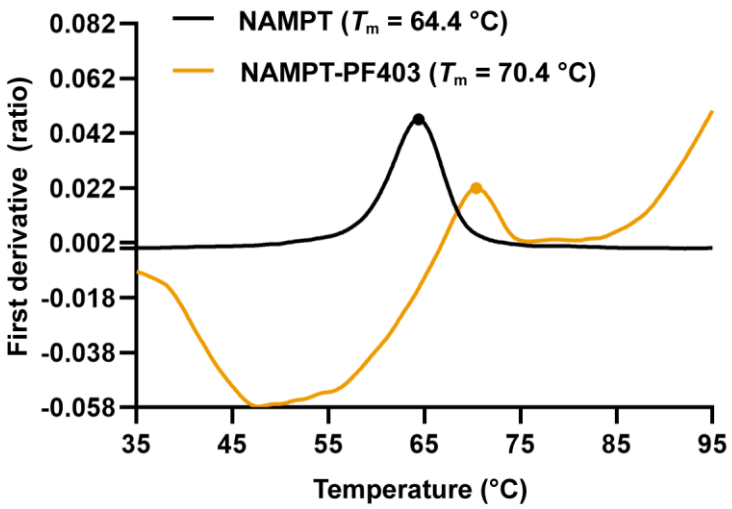

Figure S4 Binding affinity analysis of PF403 for NAMPT determined by nanodifferential scanning fluorescence (nanoDSF).

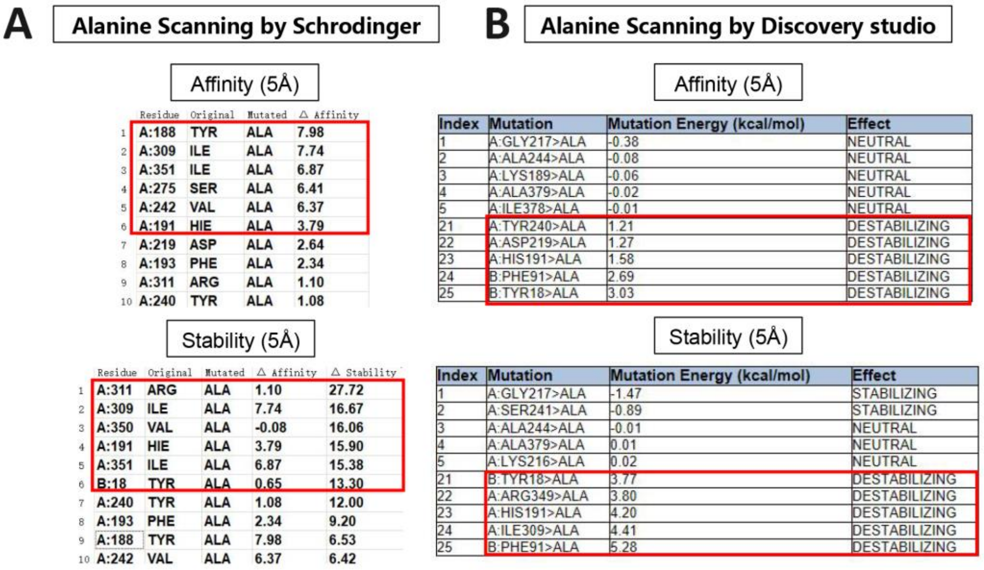

A — Affinity (5Å)

	Residue	Original	Mutated	Δ Affinity
1	A:188	TYR	ALA	7.98
2	A:309	ILE	ALA	7.74
3	A:351	ILE	ALA	6.87
4	A:275	SER	ALA	6.41
5	A:242	VAL	ALA	6.37
6	A:191	HIE	ALA	3.79
7	A:219	ASP	ALA	2.64
8	A:193	PHE	ALA	2.34
9	A:311	ARG	ALA	1.10
10	A:240	TYR	ALA	1.08

A — Stability (5Å)

	Residue	Original	Mutated	Δ Affinity	Δ Stability
1	A:311	ARG	ALA	1.10	27.72
2	A:309	ILE	ALA	7.74	16.67
3	A:350	VAL	ALA	-0.08	16.06
4	A:191	HIE	ALA	3.79	15.90
5	A:351	ILE	ALA	6.87	15.38
6	B:18	TYR	ALA	0.65	13.30
7	A:240	TYR	ALA	1.08	12.00
8	A:193	PHE	ALA	2.34	9.20
9	A:188	TYR	ALA	7.98	6.53
10	A:242	VAL	ALA	6.37	6.42

B — Affinity (5Å)

Index	Mutation	Mutation Energy (kcal/mol)	Effect
1	A:GLY217>ALA	-0.38	NEUTRAL
2	A:ALA244>ALA	-0.08	NEUTRAL
3	A:LYS189>ALA	-0.06	NEUTRAL
4	A:ALA379>ALA	-0.02	NEUTRAL
5	A:ILE378>ALA	-0.01	NEUTRAL
21	A:TYR240>ALA	1.21	DESTABILIZING
22	A:ASP219>ALA	1.27	DESTABILIZING
23	A:HIS191>ALA	1.58	DESTABILIZING
24	B:PHE91>ALA	2.69	DESTABILIZING
25	B:TYR18>ALA	3.03	DESTABILIZING

B — Stability (5Å)

Index	Mutation	Mutation Energy (kcal/mol)	Effect
1	A:GLY217>ALA	-1.47	STABILIZING
2	A:SER241>ALA	-0.89	STABILIZING
3	A:ALA244>ALA	-0.01	NEUTRAL
4	A:ALA379>ALA	0.01	NEUTRAL
5	A:LYS216>ALA	0.02	NEUTRAL
21	B:TYR18>ALA	3.77	DESTABILIZING
22	A:ARG349>ALA	3.80	DESTABILIZING
23	A:HIS191>ALA	4.20	DESTABILIZING
24	A:ILE309>ALA	4.41	DESTABILIZING
25	B:PHE91>ALA	5.28	DESTABILIZING

Figure S5 (A) The results of alanine scan by Schrödinger software. (B) The results of alanine scan by Discovery Studio software.

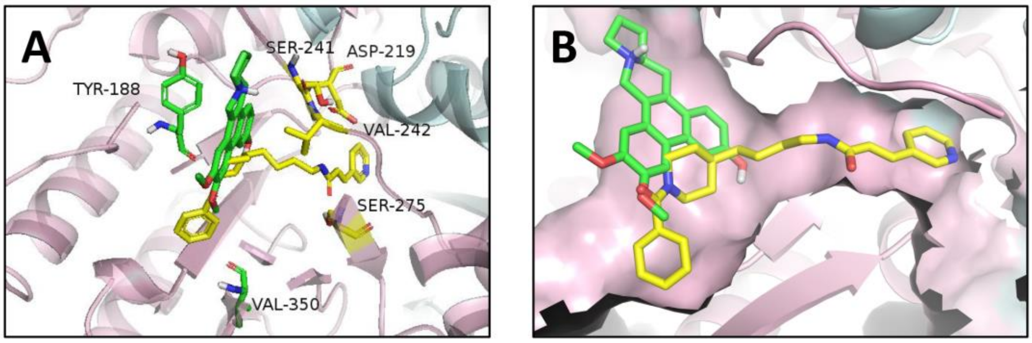

Figure S6 (A) The overlapping pattern of PF403 and FK866 bonded to NAMPT. (B) The binding posture of PF403 and FK866 in protein cavities. The green stick is PF403, and the yellow is FK866.

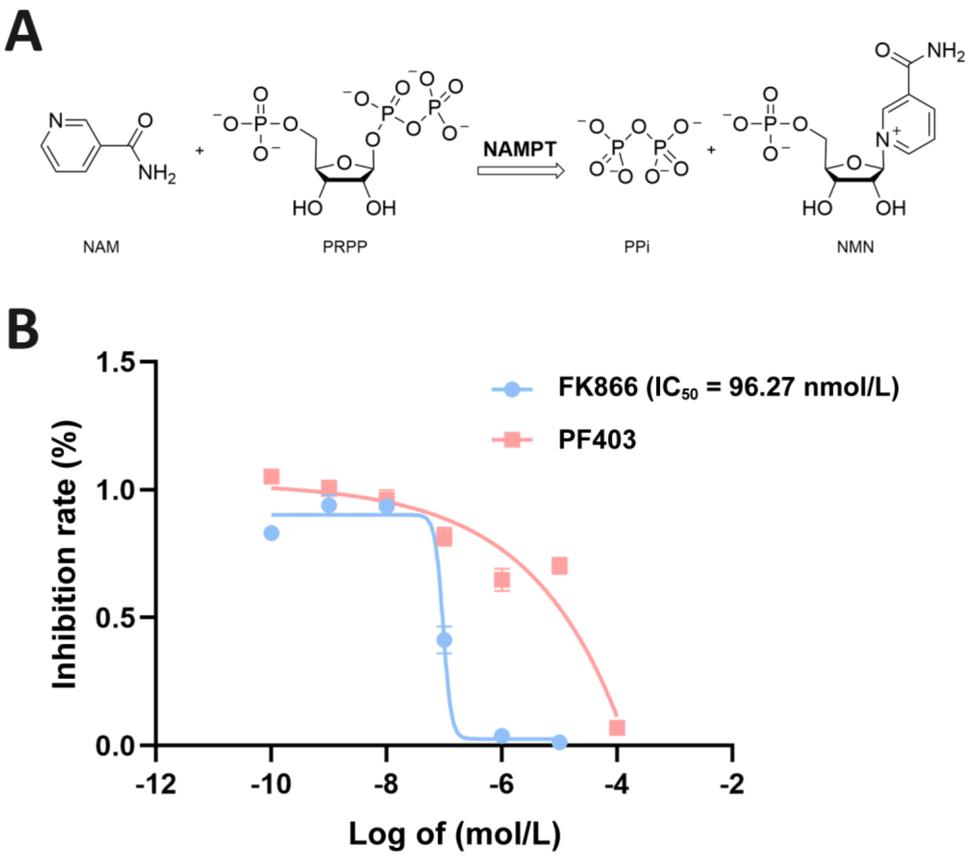

Figure S7 (A) Reaction schematic of NAMPT catalytic activity. (B) The direct inhibiting effect of PF403 and FK866 on NAMPT activity (n = 2). All data are expressed as the mean ± SD.

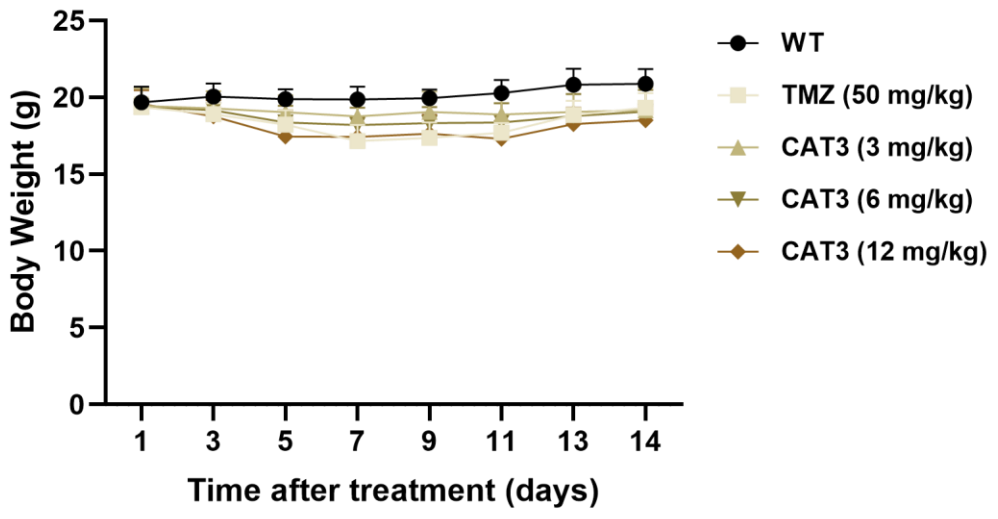

Figure S8 Body weight of mice from different treatment groups for *in vivo* efficacy evaluation experiments of CAT3 (n = 12). All data are expressed as the mean ± SD.

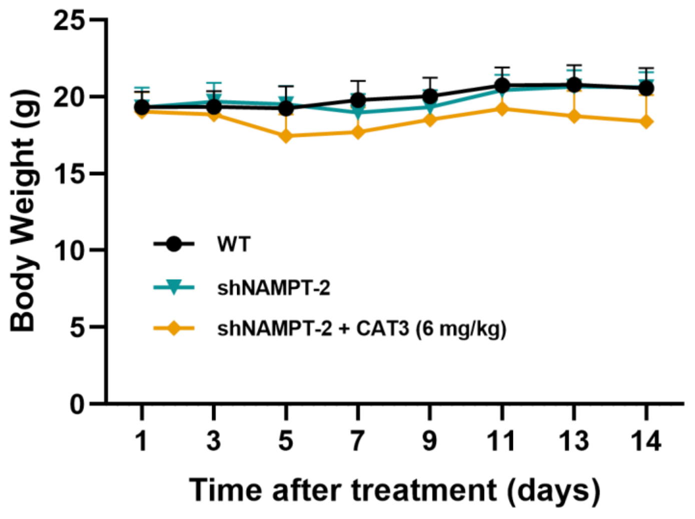

Figure S9 Body weight during 1–14 days from different treatment groups (n = 12). All data are expressed as the mean ± SD.

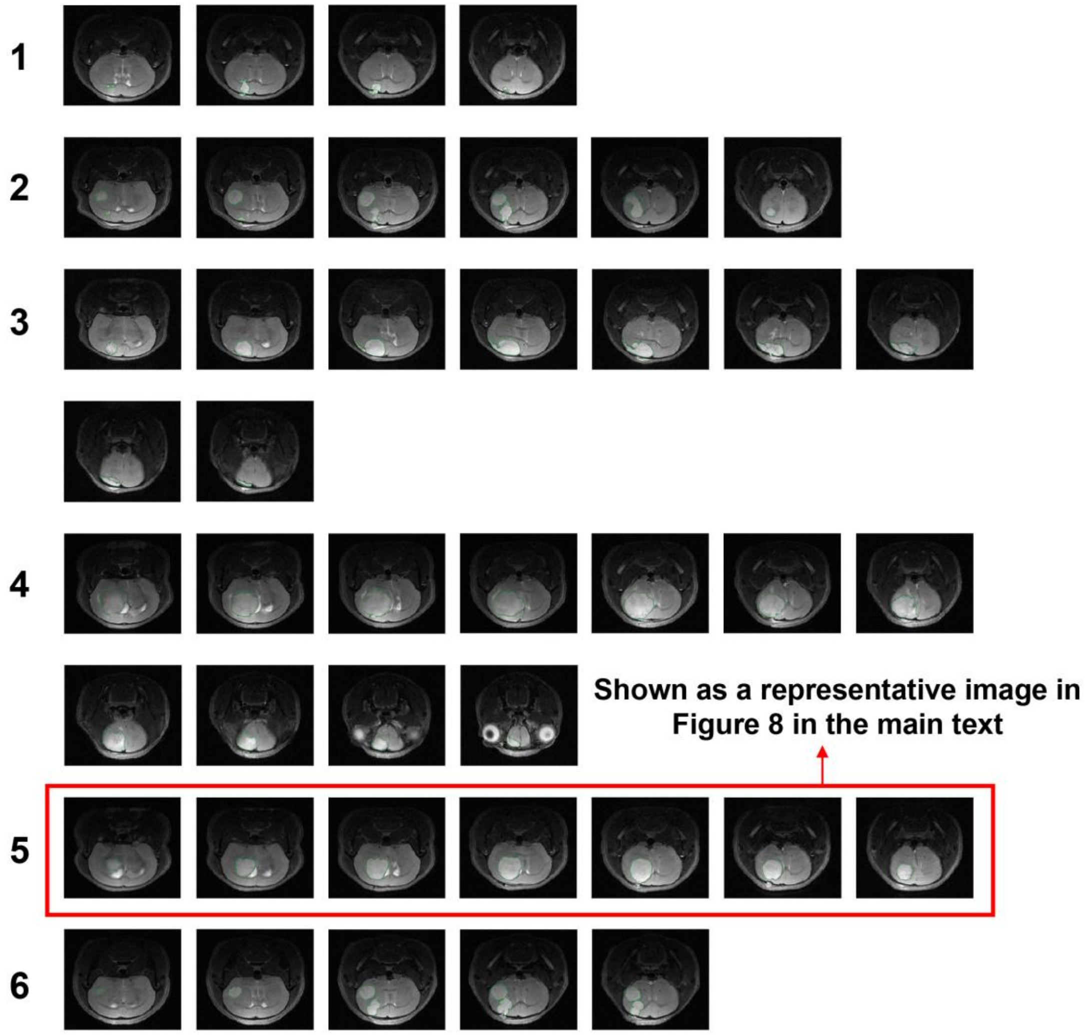

----------Not completed, see next page.

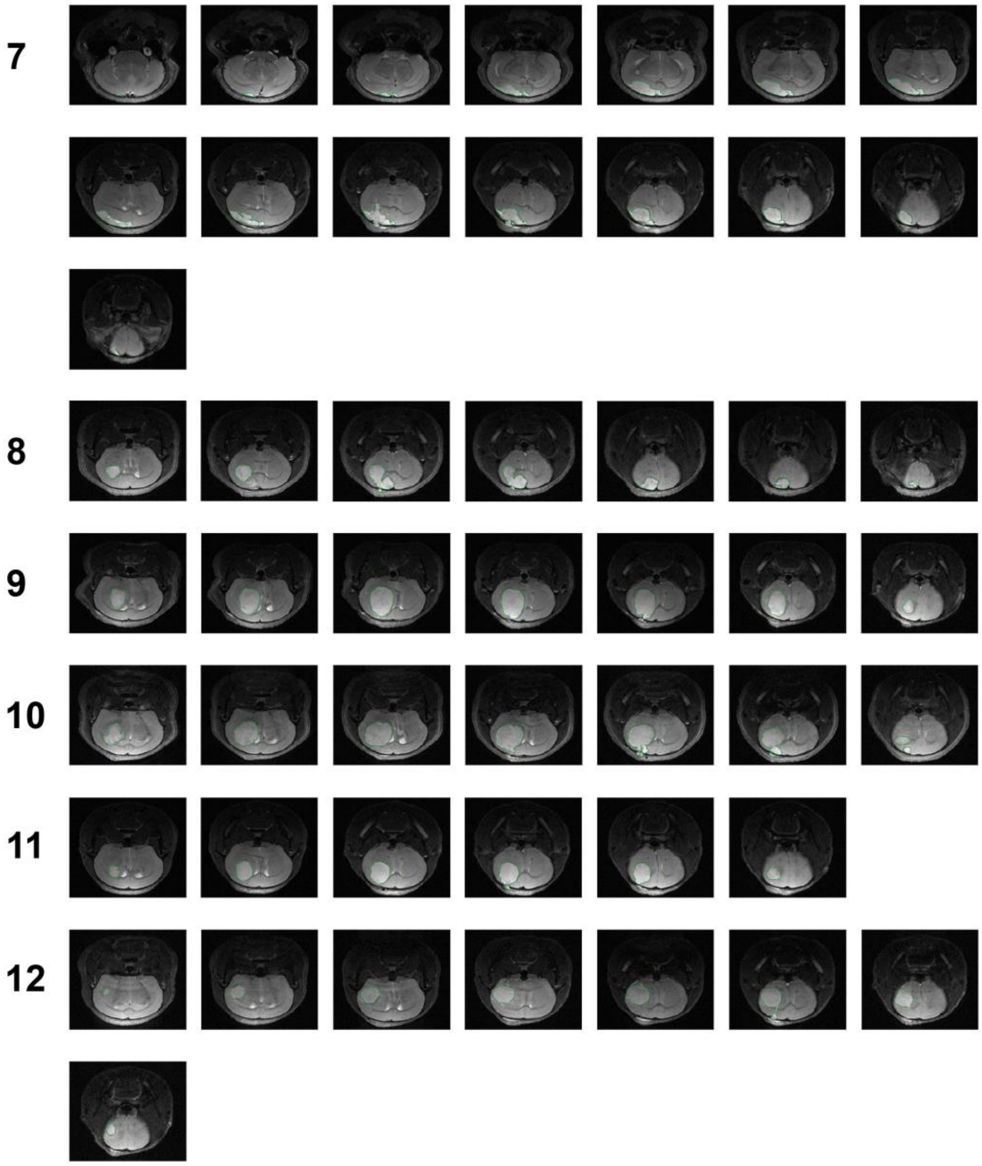

----------Not completed, see next page.

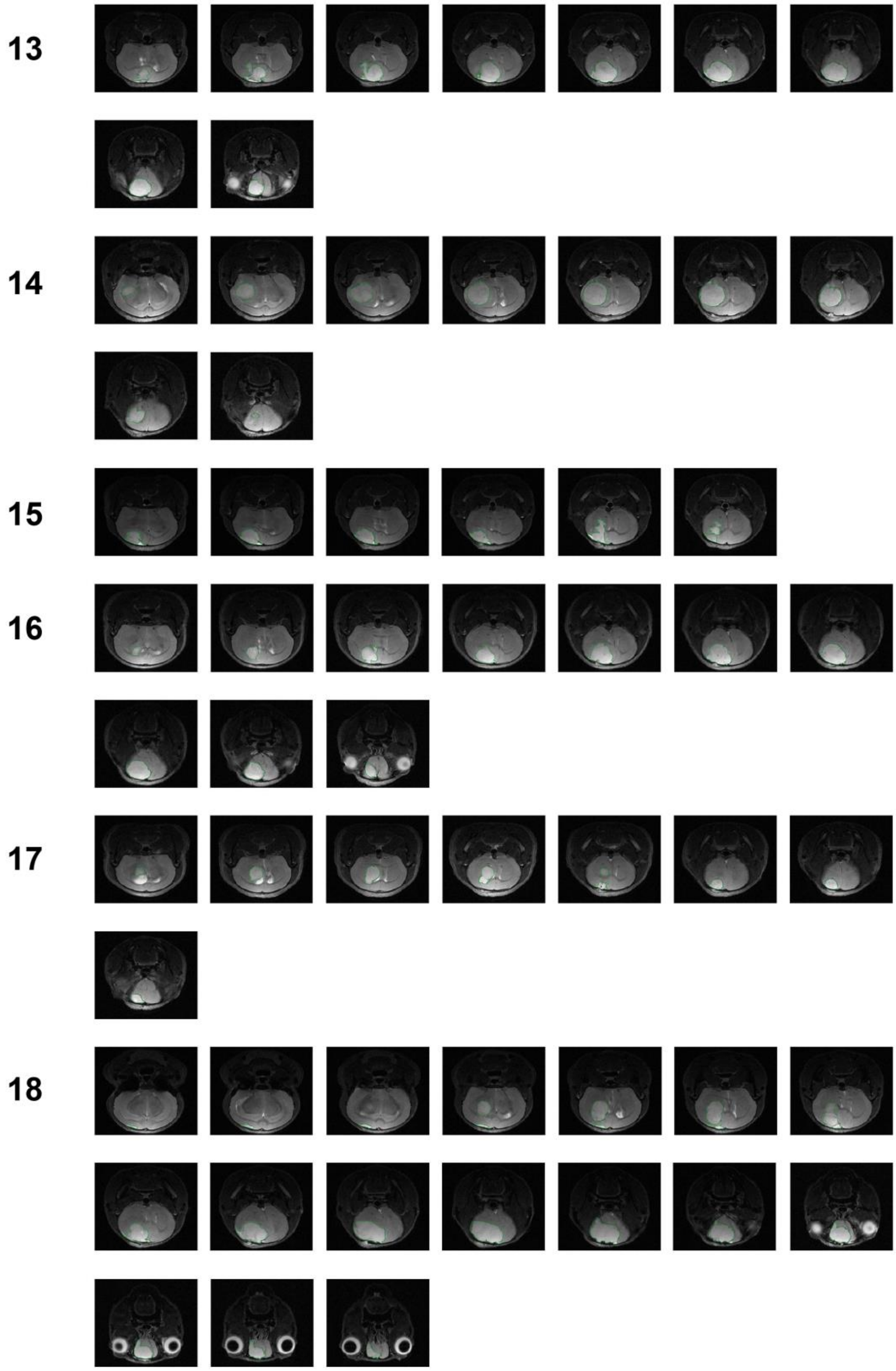

----------Not completed, see next page.

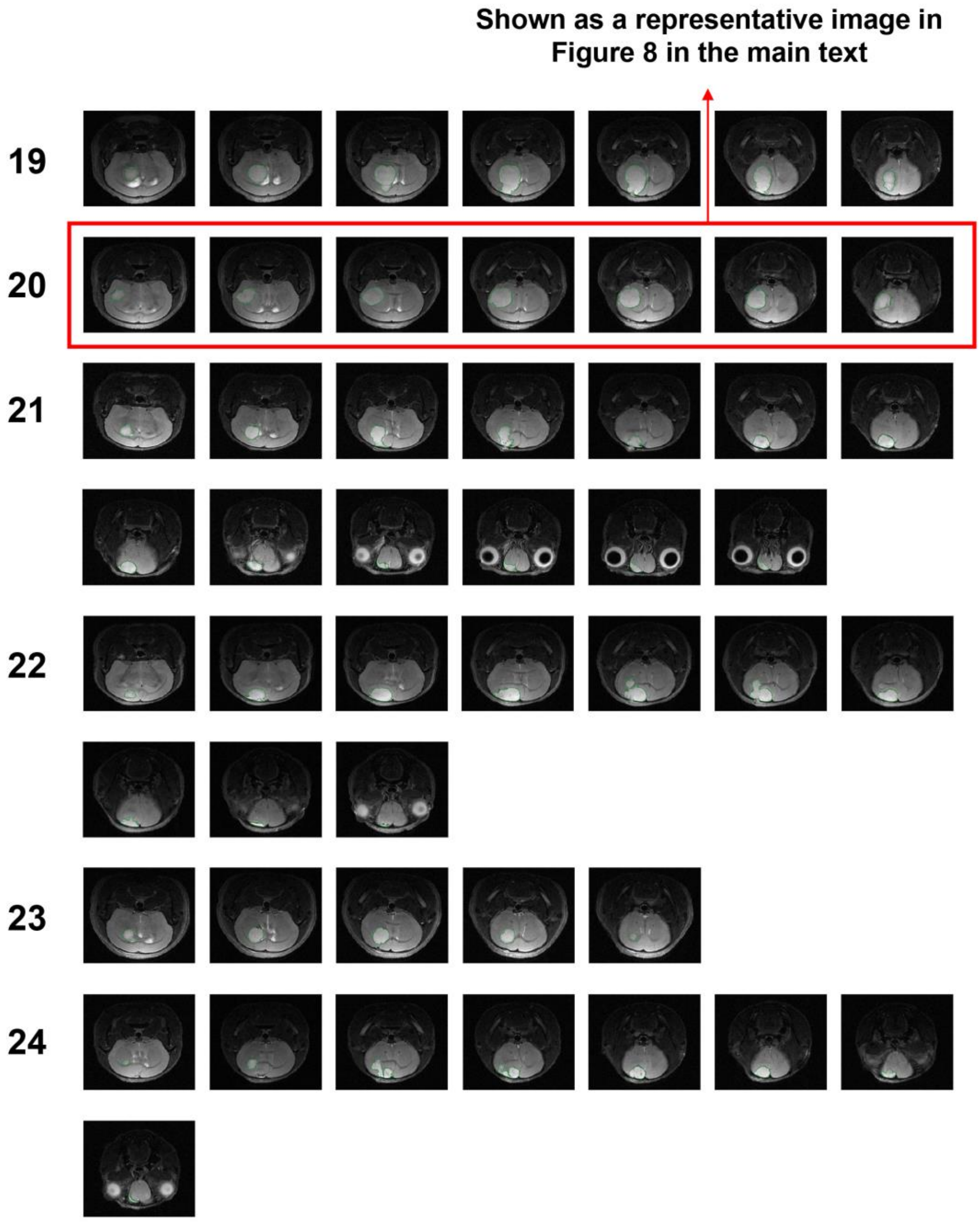

Figure S10 All T2-weighted enhanced MRI images of Orthotopic glioma model mice (1–12: Vehicle control 13–24: CAT-6mg/kg, *n* = 12).

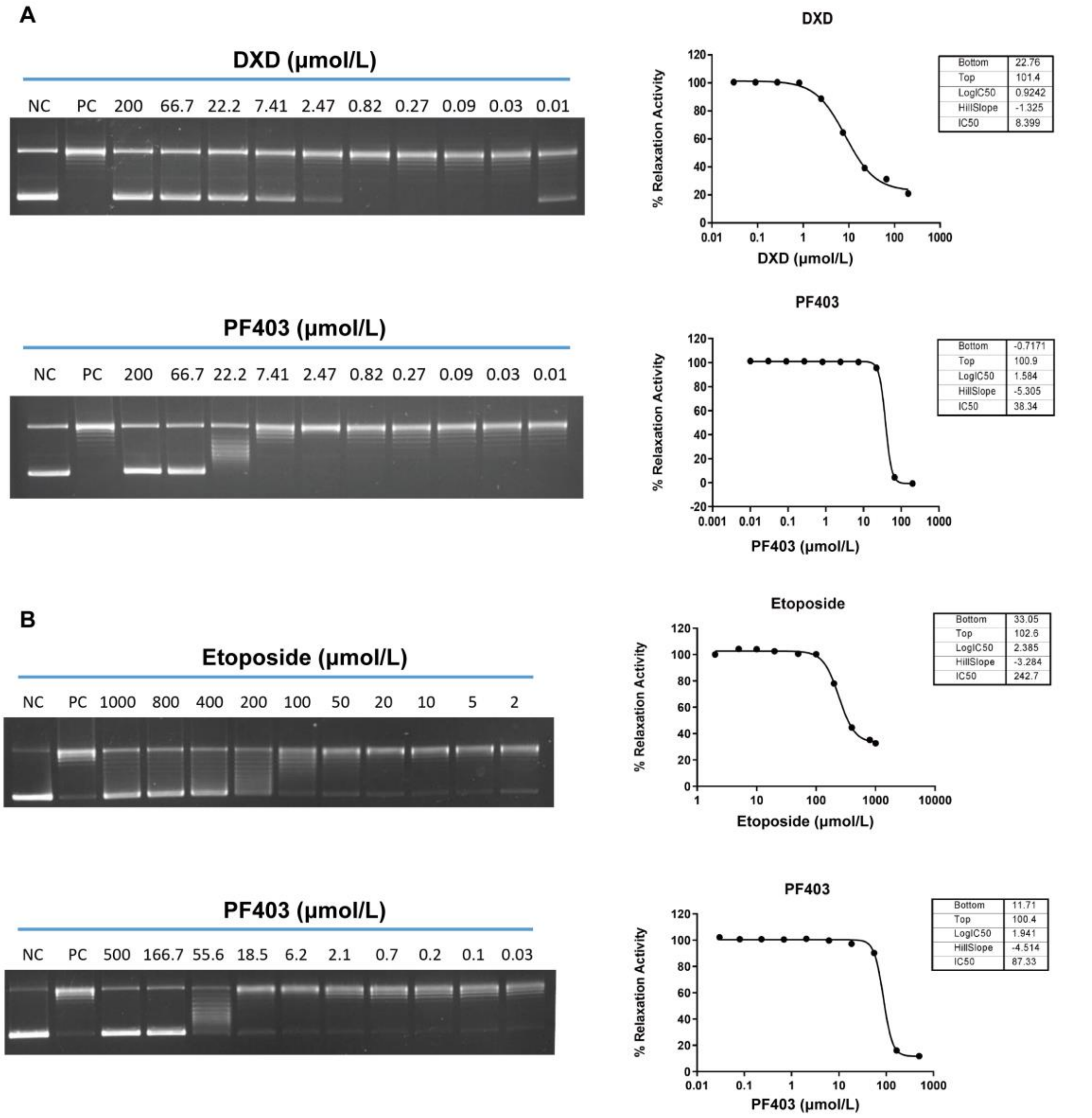

Figure S11 (A) The inhibitory activity of PF403 on topoisomerase 1 with camptothecin derivative DXD as a positive control. (B) The inhibitory activity of PF403 on topoisomerase 2 with Etoposide as a positive control.

Supporting Tables

Table S1 Screening condition parameters for protein thermal stability curves.

Index	Specific conditions
1	R^2 > 0.8 or fitted curves for DMSO and PF403 treatment.
2	Plateau of < 0.3 for curves.
3	Steepest slope of protein melting curves in paired set of DMSO and PF403 treated conditions < –0.06.
4	Melting point difference for each protein between both DMSO replicates < 1.5 ℃.
5	One of the *P* values for the two replicate experiments is < 0.1 and the other is < 0.05.
6	Melting point shifts for both paired replicates (PF403 *vs* DMSO) have the same direction.
7	Melting point difference PF403 *vs* DMSO > DMSO1 *vs* DMSO2.

Table S2 PF403 binding proteins identified by ProSAR.

Accession	Score	Repetition	*P*-value (–log10)	ΔT_m	Group1_R^2	Group2_R^2	Group1_Tm	Group2_Tm	min_Slope
NAMPT	4.882	Rep1	3.424	6.46	0.915	0.868	60.53	66.99	–0.166
		Rep2	3.014	5.52	0.975	0.975	61.47	66.99	–0.107
SAP30BP	3.036	Rep1	1.712	4.23	0.919	0.974	55.55	59.78	–0.065
		Rep2	2.806	5.26	0.928	0.83	58.5	63.76	–0.096
TP53BP1	1.673	Rep1	1.078	3.06	0.904	0.957	54.77	57.83	–0.1
		Rep2	1.612	3.49	0.857	0.855	56.05	59.54	–0.683
RPL8	1.424	Rep1	1.14	3.19	0.85	0.882	53.42	56.61	–0.075
		Rep2	1.353	3.01	0.926	0.822	55.37	58.38	–0.247

Table S3 Data collection and refinement statistics of cocrystal structure (molecular replacement).

NAMPT-PF403	Crystal 1 name
Data collection	
Space group	P 1 21 1
Cell dimensions	
a, *b*, *c* (Å)	60.93 106.42 83.00
α, β, γ (°)	90 96.37 90
Resolution (Å)	41.25 - 1.86 (1.93 - 1.86)
R_{sym} or R_{merge}	0.07574 (0.6836)
I / σI	12.49 (2.50)
Completeness (%)	99.96 (99.84)
Redundancy	4.2 (4.2)
Refinement	
Resolution (Å)	1.86
No. reflections	87811 (8773)
R_{work}/R_{free}	0.16/0.19
No. atoms	
Protein	7580
Ligand/ion	80
Water	678
B-factors	24.38
Protein	23.60
Ligand/ion	31.20
Water	32.32
R.m.s. deviations	
Bond lengths (Å)	0.014
Bond angles (°)	1.64

Glossary

ABPP	activity-based protein profiling
BBB	blood–brain barrier
CAT	(+)-Deoxytylophorinine
CAT3	(13a*S*)-3-Pivaloyloxyl-6,7-dimethoxyphenanthro[9,10-*b*] indolizidine
CCK-8	cell counting kit-8
CETSA	cellular thermal shift assay
DARTS	drug affinity responsive target stability
eNAMPT	extracellular NAMPT
FBS	Foetal bovine serum
FK866	(*E*)-Daporinad
HEBP1	heme-binding protein 1
iNAMPT	intracellular NAMPT
ITC	isothermal Titration Calorimetry
MD	molecular dynamics
MOI	multiplicity of infection
mOS	median overall survival
MST	microscale thermophoresis
NAD^+	nicotinamide adenine dinucleotide
NAM	nicotinamide
NAMPT	nicotinamide phosphoribosyl transferase
nanoDSF	nanodiometric scanning fluorescence
NOP14	nucleolar protein 14
OD	optical density
PBS	phosphate-buffered saline
PF403	(13a*S*)-3-hydroxyl-6,7-dimethoxyphenanthro[9,10-*b*]indolizidine
PFKL	phosphofructokinase-1 liver type
PISA	proteome integral solubility alteration
RMSD	root mean square deviation
RMSF	root mean square fluctuation
SP	standard precision
SPC	simple point charge
SPR	surface plasmon resonance
SPROX	stability of proteins from rates of oxidation
Tm	melting temperature
TMT10	tandem mass tag 10
TMZ	temozolomide
TOP1	topoisomerase 1
TOP2	topoisomerase 2
TPP	thermal proteome profiling
TPP-TR	thermal proteome profiling-temperature range
WT	wild-type
XP	extra precision
XRD	X-ray diffraction
